# Expression and analysis of the SAM-dependent RNA methyltransferase Rsm22 from *Saccharomyces cerevisiae*

**DOI:** 10.1107/S2059798321004149

**Published:** 2021-05-19

**Authors:** Jahangir Alam, Farah Tazkera Rahman, Shiv K. Sah-Teli, Rajaram Venkatesan, M. Kristian Koski, Kaija J. Autio, J. Kalervo Hiltunen, Alexander J. Kastaniotis

**Affiliations:** a Faculty of Biochemistry and Molecular Medicine, University of Oulu, Aapistie 7B, FIN-90220 Oulu, Finland; b Biocenter Oulu, Aapistie 5A, FIN-90220 Oulu, Finland

**Keywords:** mitochondrial ribosomes, methyltransferases, Rsm22, crystallization, SAXS structure

## Abstract

The *Saccharomyces cerevisiae* Rsm22 protein (Sc-Rsm22), encoded by the nuclear *RSM22* (systematic name *YKL155c*) gene, is a distant homologue of Rsm22 from *Trypanosoma brucei* (Tb-Rsm22) and METTL17 from mouse (Mm-METTL17). All three proteins have been shown to be associated with mitochondrial gene expression, and Sc-Rsm22 has been documented to be essential for mitochondrial respiration. The Sc-Rsm22 protein comprises a polypeptide of molecular weight 72.2 kDa that is predicted to harbor an N-terminal mitochondrial targeting sequence. The precise physiological function of Rsm22-family proteins is unknown, and no structural information has been available for Sc-Rsm22 to date. In this study, Sc-Rsm22 was expressed and purified in monomeric and dimeric forms, their folding was confirmed by circular-dichroism analyses and their low-resolution structures were determined using a small-angle X-ray scattering (SAXS) approach. The solution structure of the monomeric form of Sc-Rsm22 revealed an elongated three-domain arrangement, which differs from the shape of Tb-Rsm22 in its complex with the mitochondrial small ribosomal subunit in *T. brucei* (PDB entry 6sg9). A bioinformatic analysis revealed that the core domain in the middle (Leu117–Asp462 in Sc-Rsm22) resembles the corresponding region in Tb-Rsm22, including a Rossmann-like methyltransferase fold followed by a zinc-finger-like structure. The latter structure is not present in this position in other methyltransferases and is therefore a unique structural motif for this family. The first half of the C-terminal domain is likely to form an OB-fold, which is typically found in RNA-binding proteins and is also seen in the Tb-Rsm22 structure. In contrast, the N-terminal domain of Sc-Rsm22 is predicted to be fully α-helical and shares no sequence similarity with other family members. Functional studies demonstrated that the monomeric variant of Sc-Rsm22 methylates mitochondrial tRNAs *in vitro*. These data suggest that Sc-Rsm22 is a new and unique member of the RNA methyltransferases that is important for mitochondrial protein synthesis.

## Abstract 2

Rsm22-family proteins are conserved putative SAM-dependent methyltransferases with important functions in mitochondrial translation. Here, the results of a comparative bioinformatics analysis of Rsm22-type proteins are presented, the expression, biophysical characterization and crystallization of *Saccharomyces cerevisiae* Rsm22 are reported, a low-resolution SAXS structure of the protein is revealed, and SAM-dependent RNA methyl transferase activity of the protein is demonstrated.

## 1. Introduction   

While it has been recognized that the roles of mitochondria in eukaryotic cells far exceed the well established function of these organelles in cellular energy metabolism, ATP production by oxidative phosphorylation (OXPHOS) remains one of the pivotal tasks of mitochondria in most eukaryotes. In order to carry out OXPHOS, mitochondria must maintain a functional respiratory chain (Murphy *et al.*, 2016 ▸). Most of the proteins required for mitochondrial function are encoded by the nuclear genes and are imported into the mitochondria by an intricate machinery. However, the intramitochondrial expression of a few key proteins is required for respiring mitochondria. This set of proteins invariably contains core respiratory-chain components, which are possibly too hydrophobic to be efficiently imported and assembled (Johnston & Williams, 2016 ▸). Therefore, mitochondria have retained a pared-down genome encoding these polypeptides as well as the RNAs essential for mitochondrial gene expression. The most conserved mitochondrial DNA-encoded proteins assemble together with imported protein subunits in a highly controlled fashion to form the functional respiratory chain (Wallace, 2007 ▸). In order to be able to express the mitochondrially encoded proteins, the organelles harbor a complete transcription and translation machinery. Although the structures of yeast and mammalian mitochondrial ribosomes have been determined (Desai *et al.*, 2017 ▸; Sharma *et al.*, 2003 ▸), there are still many open questions concerning their assembly and function.

**Figure 1:**
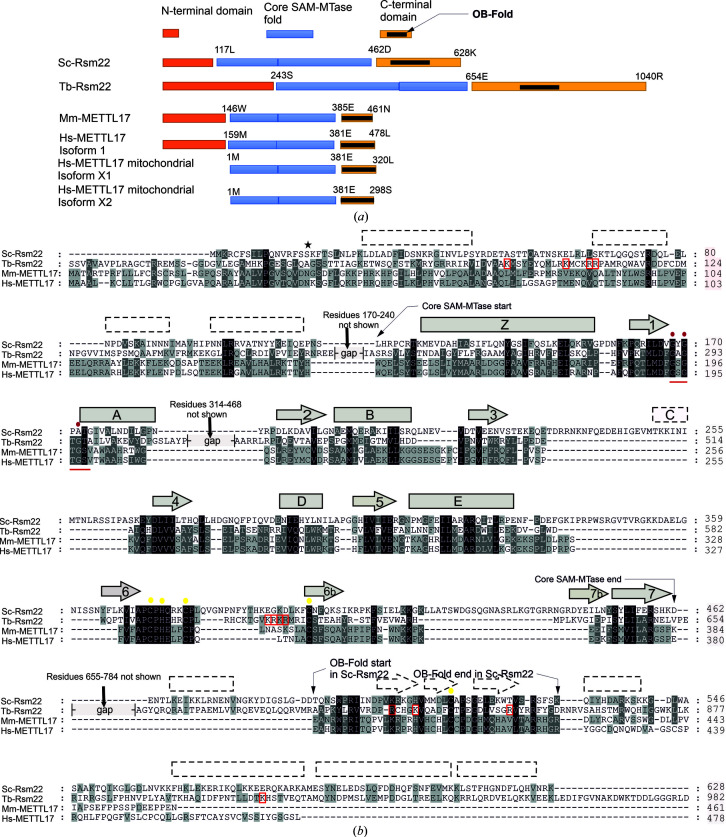
Bioinformatic analysis of Rsm22-family proteins. (*a*) Predicted domain structure of the Rsm22 family. The SAM-binding domain and substrate-binding domain together comprise a Rossmann-like SAM-MTase fold, which is located in the middle. The N-termini are highly variable. There is an extended N-­terminal domain preceding the SAM-binding domain in Tb-Rsm22. The N-terminal domain is absent in Hs-METTL17 isoforms X1 and X2. The C-­terminal domain is also variable between the family members, being shortest in Hs-METTL17, but contains a relatively conserved OB-motif in all family members. (*b*) Sequence alignment of *S. cerevisiae* Rsm22 (Sc-Rsm22) with Rsm22 from *T. brucei* (Tb-Rsm22) and METTL17 from mouse (Mm-METTL17) and human (Hs-METTL17). The predicted secondary-structure elements of Sc-Rsm22 are shown above the sequence, with rectangles representing α-helices and arrows representing β-strands. The shaded secondary elements are those with the highest probabilities, and the prediction is based on analysis using the *Phyre*2 server (Kelley *et al.*, 2015 ▸; http://www.sbg.bio.ic.ac.uk/~phyre2/html/page.cgi?id=index) and on sequence comparison with Tb-Rsm22, the structure of which was determined by cryo-EM (Saurer *et al.*, 2019 ▸). The elements shown with dotted lines are only based on the amino-acid sequence-based prediction (by the *Phyre*2 server). The star (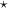) indicates the end of the predicted mitochondrial targeting sequence of Sc-­Rsm22. The MTase core fold consists of α-helices Z, A, B, C, D and E and β-strands 1–7. β-Strands 6b and 7b are unique to the Rsm22 family of methyltransferases and include the zinc-finger structure motif (zinc-binding residues are highlighted with yellow dots). The glycine-rich SAM-binding motif is also emphasized with a red line below the sequences, and the important glycines are indicated by red dots above the sequences. The positively charged residues which interact with rRNA in the *T. brucei* mitoribosome complex (Saurer *et al.*, 2019 ▸) are highlighted with red open squares in the Tb-Rsm22 sequence. An OB-fold is observed in the C-terminus of Tb-Rsm22 and is also predicted to be present in other members of the protein family.

*Saccharomyces cerevisiae* Rsm22 (Sc-Rsm22) is a nuclear-encoded protein localized to the mitochondria. Our group has found the *S. cerevisiae* *RSM22* gene to genetically interact with mitochondrial fatty-acid synthesis (mtFAS), a process that has been suggested to coordinate mitochondrial biogenesis with acetyl-CoA availability (Kursu *et al.*, 2013 ▸; Van Vranken *et al.*, 2018 ▸). Deletion of Sc-Rsm22 causes respiratory deficiency in yeast (Dimmer *et al.*, 2002 ▸). Previous studies have also found that Sc-Rsm22 physically interacts with the small subunit of the yeast mitochondrial ribosome (Saveanu *et al.*, 2001 ▸). However, Sc-Rsm22 was not found in the cryo-electron microscopy (cryo-EM) structure of the *S. cerevisiae* mitochondrial ribosome, indicating that Sc-Rsm22 might associate with the yeast mitochondrial ribosome only transiently or weakly (Desai *et al.*, 2017 ▸). *Trypanosoma brucei* Rsm22 (Tb-Rsm22), which is a distant homologue of Sc-Rsm22 (Fig. 1 ▸), has a role in the assembly of the mitochondrial ribosome in this parasite (Týč *et al.*, 2017 ▸) and has recently been found to be part of the mitoribosome complex (Saurer *et al.*, 2019 ▸). Depletion of Tb-Rsm22 leads to a compromised structure of the mitochondrial ribosomal small subunit. Loss of Tb-Rsm22 causes a severe decrease in *de novo* synthesis of mitochondrial proteins (Týč *et al.*, 2017 ▸). The mammalian homologue of Rsm22 is called methyltransferase-like protein 17 (METTL17), and has been shown to play a regulatory role in mitochondrial RNA-modification processes in mice (Shi *et al.*, 2019 ▸). Loss of mouse METTL17 (Mm-METTL17) results in a reduction in mitochondrial ribosomal RNA (mt-rRNA). Deletion of Mm-METTL17 affects the synthesis of mitochondria-encoded proteins (Shi *et al.*, 2019 ▸). It has been reported that the corresponding human METTL17 (Hs-METTL17) interacts physically and functionally with estrogen receptors in humans. Inhibition of Hs-METTL17 results in a decrease in the level of mRNA and proteins encoded by estrogen receptor target genes, and knockdown of Hs-METTL17 reduces breast cancer cell growth (Du *et al.*, 2015 ▸).

Sc-Rsm22 proteins belong to the large and diverse *S*-adenosyl-l-methionine (SAM)-dependent methyltransferase (SAM-MTase) protein family (Szczepińska *et al.*, 2014 ▸). SAM-MTases constitute a common family of enzymes which catalyze the transfer of a methyl group from SAM to N, C or O atoms within proteins, nucleic acids, small molecules and lipids (Schubert *et al.*, 2003 ▸). Sc-Rsm22 and its homologues belong to the class I SAM-MTases. This group of enzymes is characterized by a seven-stranded β-sheet core sandwiched by six α-helices, which is very similar to the well known Rossmann fold (Martin & McMillan, 2002 ▸; Szczepińska *et al.*, 2014 ▸). The main difference between the class I MTase Rossmann fold-like structure and the classical Rossmann fold is the last β-strand β7 of the core β-sheet, which is antiparallel and is not present in the latter. At the end of β-strand 1 is a conserved glycine-rich (Gly-*X*-Gly-*X*-Gly) region, which is important for SAM binding. The substrate of Sc-Rsm22 has not been reported. According to previous bioinformatics studies of the yeast ‘methyltransferome’, Sc-Rsm22 was initially predicted to be a protein MTase (Wlodarski *et al.*, 2011 ▸). However, more recent *in silico* work has classified yeast Rsm22 as an RNA MTase (Szczepińska *et al.*, 2014 ▸). In the recently published cryo-EM structure of the *T. brucei* mitoribosome (Saurer *et al.*, 2019 ▸) Tb-Rsm22 interacts with several proteins, and also makes a contact with rRNA, but the function and the substrate of Tb-Rsm22 were not revealed by this structure.

Previous reports indicated that the rRNA component of the small ribosomal subunit in yeast is not methylated (Klootwijk *et al.*, 1975 ▸). In contrast, mitochondrial tRNAs, like tRNAs in general (Machnicka *et al.*, 2014 ▸), carry multiple methylation modifications, the methyltransferases responsible for many of which are not known. Very often, tRNA-modifying enzymes and tRNA modifications are highly conserved throughout multiple domains of life (Swinehart *et al.*, 2013 ▸). Many human diseases such as neurological and mitochondrial disorders, as well as cancer and type 2 diabetes, are linked to mitochondrial tRNA (mt-tRNA) modifications (Torres *et al.*, 2014 ▸; Boczonadi *et al.*, 2018 ▸). The tRNA methyltransferases identified to date mostly belong to the class I or class IV (SPOUT) SAM-MTases (McKenney *et al.*, 2017 ▸). The known tRNA methyltransferases are a structurally very diverse group of enzymes that share a common SAM-MTAse core fold but are supplemented with variable domains required for tRNA binding (McKenney *et al.*, 2017 ▸). Only a few structures of tRNA MTases have been published. Some of these have been determined in complex with SAM and tRNA, with one example being the archaeal Trm5 (Goto-Ito *et al.*, 2009 ▸).

In the present study, employing bioinformatics, enzymological and structural methods, we demonstrate that Sc-Rsm22 is a class I RNA MTase which is structurally distinct from the trypanosomal homologue. We report the expression, purification and solution structure of Sc-Rsm22. The solution structure of the protein from small-angle X-ray scattering (SAXS) studies shows Sc-Rsm22 as an elongated monomer which can also further dimerize to form a homodimer. The possible structure and key residues for substrate recognition by a proposed OB-fold are also discussed. Our *in vitro* enzymatic assay shows that mitochondrial tRNAs are accepted as substrates for methylation by monomeric Sc-Rsm22.

## 2. Materials and methods   

### 2.1. Yeast DNA isolation from *S. cerevisiae*   

The genomic DNA of *S. cerevisiae* strain W1536 5B was isolated using the protocol developed by Hoffman & Winston (1987 ▸). Yeast cells were grown in a 10 ml culture overnight in an appropriate selective (synthetic complete glucose/SCD; Formedium) medium to saturation. The cells were then harvested by centrifugation at 4200*g* for 5 min and resuspended in water. The cells were transferred to a 1.5 ml Eppendorf tube and centrifuged again at 17 900*g* for 2 min. The supernatant was discarded and the pellet was resuspended in the residual liquid. 0.2 ml 2% Triton X-100, 1% SDS, 100 m*M* NaCl, 10 m*M* Tris pH 8.0 and 1 m*M* EDTA were added. 0.2 ml phenol–chloroform–isoamyl alcohol (25:24:1) and 0.3 g acid-washed glass beads (0.45–0.52 nm diameter) were added in the last step and vortexed thoroughly for 3–4 min. 0.3 ml Tris–EDTA (TE) was added to the mixture, which was centrifuged at 17 900*g* for 5 min. The aqueous layer (containing the DNA) was transferred to a new Eppendorf tube and 1.0 ml 100% ethanol was added. The tube contents were mixed by inverting and then centrifuged at 17 900*g* for 2 min. The supernatant was discarded and the pellet was resuspended in 0.4 ml TE plus 30 µg RNase. The solution was incubated for 5 min at 37°C and 10 µl 4 *M* ammonium acetate and 1 ml 100% ethanol were then added; the tube contents were mixed by inverting and centrifuged at 17 900*g* for 2 min. The pellet containing the DNA was resuspended in 50 µl TE.

### 2.2. Molecular cloning of *S. cerevisiae* *RSM22*   

The *RSM22* open reading frame encodes 628 amino-acid residues. Analysis of the Sc-Rsm22 protein sequence using *MitoProt II* and *Target P* 1.1 (Emanuelsson *et al.*, 2000 ▸, 2007 ▸) indicated that the first 15 or 16 amino acids constitute a cleavable N-terminal mitochondrial targeting signal. To produce native mature Sc-Rsm22 for purification, a construct (UniProt ID P36056; construct P36056; MA-S17–K628-HHHHHH) for the overexpression of six-histidine-tagged Sc-Rsm22 protein in *Escherichia coli* was designed. *RSM22* was amplified by polymerase chain reaction (PCR) using forward and reverse primers introducing an NcoI site and an XhoI site, respectively, for cloning into the NcoI/XhoI sites of vector pET-23d (Novagen/EMD Biosciences). Primer sequences are listed in Supplementary Table S1. Use of the NcoI site introduces an ATG start codon followed by a G. To complete the codon and ensure that the *RSM22* ORF was translated in frame, one C and one T residue were added. Hence, the first codon following the translation start site is GCT, encoding alanine. The vector introduces six histidines at the C-terminus of Sc-Rsm22, followed by a stop codon. Total DNA isolated from the W1536 5B yeast strain (Kastaniotis *et al.*, 2004 ▸) was used as a template and PCR was carried out using a PCR kit from Thermo Fisher Scientific in a PTC-100 Peltier effect thermal cycler (MJ Research). The plasmid and PCR products were restriction-digested using NcoI and XhoI enzymes (New England Biolabs) and subjected to agarose gel electrophoresis to separate the digested fragments. The appropriately sized plasmid fragment and digested PCR products were purified with a purification kit (NucleoSpin Gel and PCR Clean-up). A Thermo Fisher Scientific DNA ligation kit was used for the ligation of restriction-digested PCR product and plasmid fragments.

### 2.3. Expression and purification   

The pET-23d-*RSM22* expression vector was transformed into *E. coli* strain C41 (Sigma–Aldrich). For expression of the Sc-Rsm22 protein, the transformed cells were precultured in 10 ml LB medium supplemented with 100 mg l^−1^ ampicillin at 37°C overnight. The next day, 5 ml of the preculture was added to 1 l autoinduction medium (55 g powdered medium from Formedium and 10 ml 100% glycerol in a 1 l final volume) supplemented with 100 mg l^−1^ ampicillin. The cells were grown at 37°C until the OD_600_ reached 1.5; the culture was then switched to a lower temperature (18°C) and incubation was continued overnight for overexpression in autoinduction medium. After overexpression, the cells were harvested using a JS4.2 rotor (4000 rev min^−1^, 45 min, 4°C), after which the supernatant was discarded and the cells were resuspended in lysis solution (40 m*M* Tris pH 7.5, 500 m*M* NaCl, 5% glycerol) with one EDTA-free protease-inhibitor tablet (Roche) per 50 ml. 5 m*M* MgCl_2_, 0.1 mg ml^−1^ lysozyme and 0.025 mg ml^−1^ DNase I (final concentration) were added and incubated at room temperature for 20 min. Finally, cell lysis (50 ml) was performed by sonication with a digital sonifier (Branson) on ice (15% amplitude for 3 min using cycles of 15 s on and 30 s off to avoid overheating the sample). The total cell lysate was then subjected to centrifugation for 50 min at 15 000 rev min^−1^ using an SS-34 rotor at 4°C. The cell pellet and supernatant were separated. 5 m*M* imidazole (final concentration) was added to the supernatant followed by filtration with a 0.45 µm filter. An Ni–NTA immobilized metal-affinity chromatography (IMAC) column was prepared with 2 ml 50% Ni–NTA agarose (Qiagen). The matrix was washed with 20 ml distilled water followed by equilibration with 10 ml initial wash solution (40 m*M* Tris pH 7.5, 500 m*M* NaCl, 5% glycerol, 5 m*M* imidazole). Filtrated protein solution was applied onto the equilibrated column. The column was consecutively washed with 30 ml wash solution with increasing imidazole concentrations (5, 20 and 25 m*M* imidazole) and each flowthrough fraction was collected. Finally, the protein was eluted three times using 3 ml elution solution (40 m*M* Tris pH 7.5, 500 m*M* NaCl, 5% glycerol, 333 m*M* imidazole).

In the next step, 22% ammonium sulfate (final concentration) was slowly added to the eluted protein and the solution was stirred at 4°C for 30 min. The precipitated protein was collected by centrifugation and dialyzed against 40 m*M* Tris pH 7.5, 500 m*M* NaCl, 5% glycerol, which was the optimal buffer based on our dialysis and dynamic light-scattering (DLS) experiments (see Section 2.6). Final purification was performed by size-exclusion chromatography (SEC) using this optimal buffer. The protein sample was filtrated with an 0.2 µm filter before application onto the column. SEC was carried out using either a Superdex 200 10/300 GL column or a HiLoad 16/600 Superdex 200 column (GE Healthcare) with either BioLogic DuoFlow (Bio-Rad) or ÄKTApurifier (GE Healthcare) protein-purification equipment at 5–10°C.

An attempt to use cation-exchange chromatography as an additional purification step was unsuccessful. This step required the salt concentration of the solvent to be changed. We tested the solubility of the protein at different pH values and salt concentrations. We noticed precipitation and aggregation of the purified protein during the change from the initial solvent to another solvent with low salt concentration. Overnight dialysis of Sc-Rsm22 was performed for buffer exchange. We tested four buffer conditions varying in pH value and salt concentration (Supplementary Table S2). Soluble Sc-Rsm22 was found in one condition (40 m*M* Tris pH 7.5, 500 m*M* NaCl, 5% glycerol), which was the initial purification condition. Further DLS experiments were performed to test additional buffer conditions (Supplementary Table S3). All of the conditions showed aggregation of the protein except for the original purification condition. We thus excluded cation-exchange chromatography as a possible additional purification step and continued the purification of Sc-Rsm22 by SEC in 40 m*M* Tris pH 7.5, 500 m*M* NaCl, 5% glycerol after IMAC.

### 2.4. SDS–PAGE analysis   

Samples were separated in a 4–20% SDS–polyacrylamide gradient gel using Tris–HCl, glycine, 10% SDS buffer and running at 200 V and 400 mA for 60 min. The gel was stained for 20 min in 0.25% Coomassie Blue, 30% ethanol, 10% glacial acetic acid solution and the background stain was removed using 10% ethanol, 30% glacial acetic acid solution. The image of the Coomassie-stained gel was captured using a ChemiDoc XRS+ gel imager (Bio-Rad).

### 2.5. Mass spectrometry (MS)   

MS was used to confirm the identity of the purified protein as Sc-Rsm22. After IMAC and SEC, the solution containing the purified protein was subjected to SDS–PAGE and stained with Coomassie Blue. Protein bands were cut out of the gel, washed with water and treated with trypsin overnight. After the tryptic digestion, 2% trifluoroacetic acid was added, mixed and run on an ultrafleXtreme MALDI TOF-TOF mass spectrometer (Bruker). The data were analyzed using *Bruker BioTools* (Bruker) and *Mascot* (Matrix Science). All of the MS experiments and analysis were performed in the Biocenter Oulu Proteomics and Protein Analysis core facility.

### 2.6. Dynamic light scattering (DLS)   

Sc-Rsm22 was purified in 40 m*M* Tris pH 7.5, 500 m*M* NaCl, 5% glycerol. Overnight dialysis of purified Rsm22 was carried out with four buffer conditions (Supplementary Table S2) at 4°C. Soluble protein was only found in 40 m*M* Tris pH 7 5, 500 m*M* NaCl, 5% glycerol after dialysis. Dialyzed protein was concentrated and used in DLS experiments. Buffer solutions were prepared using a Freedom Evo pipetting robot (Tecan) for DLS experiments (Supplementary Table S3). 100 µl 0.1 mg ml^−1^ protein samples were used in 96-well plates. DLS experiments were perfomed using a DynaPro Plate Reader II and *DYNAMICS* version 7.1.7.16 (Wyatt Technology). Experiments were performed at 25°C. 20 acquisitions and images were collected.

### 2.7. Multi-angle light scattering (MALS)   

MALS experiments, online with SEC (SEC-MALS), were carried out to determine the molecular masses of the purified Sc-Rsm22 oligomers. Both Sc-Rsm22 peaks obtained from the SEC run were pooled and concentrated and were subjected to SEC-MALS analysis in 40 m*M* Tris pH 7.5, 500 m*M* NaCl, 5% glycerol (filtered with 0.1 µm filters and degassed). A Superdex 200 10/300 GL column connected to an ÄKTApurifier system was pre-equilibrated and used in the MALS procedure with a miniDAWN TREOS MALS device (Wyatt Technologies). The protein samples were passed through a refractive-index detector (RI-101; Shodex) before entering the MALS detector. Data analysis was carried out using the *ASTRA* software (Wyatt Technologies).

### 2.8. Circular-dichroism (CD) spectroscopy   

CD experiments were performed to elucidate the secondary structure and determine the thermal stability of both the monomeric and dimeric forms of Sc-Rsm22. The protein concentration of the sample was 0.1 mg ml^−1^. Sc-Rsm22 was purified in 40 m*M* Tris pH 7.5, 500 m*M* NaCl, 5% glycerol. The solvent composition in the purified protein sample was not optimal for CD measurements owing to the presence of a very high salt concentration (500 m*M* NaCl). The protein samples and solvent (blank) were diluted just before the CD measurements to avoid high salt concentrations in the CD measurements. The final solvent composition used in the experiment was 2.28 m*M* Tris pH 7.5, 28.5 m*M* NaCl, 0.28% glycerol. The pH of the solvent was checked after dilution. CD spectroscopy was performed using a Chirascan CD spectrometer (Applied Photophysics, Leatherhead, UK). CD data were collected between 280 and 190 nm at 22°C using a 0.1 cm path-length quartz cuvette. CD measurements were acquired every 1 nm with a 0.5 s integration time and were repeated three times with baseline correction. Dimeric Sc-Rsm22 was stable during data collection. We used the average of three repetitions in the analysis. However, monomeric Sc-Rsm22 showed instability after dilution. Here, we did not consider the average of the three data sets, as they deviated from each other. We used the first set of data for analysis. We then waited for 30 min and found that the diluted monomeric Sc-Rsm22 became stable. We collected ten data sets for monomeric Sc-Rsm22 and used the final data for analysis. The data were processed, including baseline subtraction, using *Chirascan Pro-Data Viewer* (Applied Photophysics) and *CDNN* (http://www.gerald-böhm.de/download/cdnn). The direct CD measurements (θ; mdeg) were converted to mean residue molar ellipticities ([θ]MR) by *Chirascan Pro-Data Viewer*. Thermal unfolding was recorded between 190 and 280 nm with a 2°C step size (from 22 to 92°C) at a 1°C min^−1^ ramp rate with ±0.2°C tolerance. The melting temperature was analyzed with *Global* 3 (Applied Photophysics) using all data recorded. The experiment was performed in the Biocenter Oulu Proteomics and Protein Analysis core facility.

### 2.9. Small-angle X-ray scattering (SAXS) data collection   

Sc-Rsm22 was purified in 40 m*M* Tris pH 7.5, 500 m*M* NaCl, 5% glycerol, 2.5 m*M* DTT for SAXS studies. Monomeric (2.9 mg ml^−1^) and dimeric (5 mg ml^−1^) Sc-Rsm22 were used separately in batch-mode experiments on the B21 beamline at Diamond Light Source (DLS), UK. Scattered X-rays at a wavelength of 1 Å (at 12.4 keV) were recorded with a PILATUS 2M detector. Buffer scattering was subtracted from the protein scattering using *SCÅTTER* (Förster *et al.*, 2010 ▸) and *PRIMUS* (Konarev *et al.*, 2003 ▸). The radius of gyration *R*_g_, forward scattering *I*_0_, maximum dimension *D*_max_ and inter­atomic distance distribution function *P*(*r*) were estimated by *SCÅTTER* and *PRIMUS* in the *GNOM* package (Svergun, 1992 ▸). *Ab initio* models of both the monomer and dimer of Sc-Rsm22 were generated by *DAMMIN* from the online SAXS cluster at EMBL, Hamburg (Svergun, 1999 ▸) using the output file from *GNOM*. The *ab initio* bead models were visualized in *PyMOL* (version 2.0; Schrödinger); the two monomer *ab initio* shapes were superimposed on the dimer *ab initio* shape by *SUPCOMB* (Kozin & Svergun, 2001 ▸) and were finally fitted manually. The Kratky plot graphs and the pair distribution curves were generated from *PRIMUS* (Konarev *et al.*, 2003 ▸) and the graphs were plotted using *Origin Pro* 9.1 (OriginLab Corporation, Northampton, Massachusetts, USA). Porod volumes were calculated using *q*-range values (7.5/*R*_g_) with *PRIMUS*. The molecular mass of the protein in solution (from both batch and online SAXS experiments) was estimated using the protein volume from the Porod invariant (MM_qp_), the *SAXMoW* tool (MoW), the empirical volume of correlation (*V*_c_) and the size and shape methods implemented in *DATTOOLS* from the *ATSAS* package (*PRIMUS*) as described previously (Hajizadeh *et al.*, 2018 ▸).

Additionally, SEC-SAXS (online SAXS) experiments were performed using purified (mixture of dimeric and monomeric) Sc-Rsm22 (2.85 mg ml^−1^). Online SAXS was carried out with a Superdex 200 5/150 column (GE Healthcare) and 40 m*M* Tris pH 7.5, 500 m*M* NaCl, 5% glycerol, 2.4 m*M* DTT solution on the BM29 beamline at ESRF. The collected data were processed similarly as in the batch-mode SAXS analysis.

### 2.10. Bioinformatics and modeling   

The initial amino-acid sequence alignment of four Rsm22-family proteins (Sc-Rsm22, Tb-Rsm22, Mm-METTL17 and Hs-METTL17) and the monomeric atomic model of Sc-Rsm22 was generated using *ClustalX* and *SWISS-MODEL* (Waterhouse *et al.*, 2018 ▸). Tb-Rsm22 from the mt-SSU assemblosome (PDB entry 6sga, chain F1) was used as the atomic model for Sc-Rsm22. This initial model was further improved using results from analysis using the *Phyre*2 server (Kelley *et al.*, 2015 ▸). *COILS* (Lupas *et al.*, 1991 ▸) was used to detect possible coiled-coil regions in the Sc-Rsm22 sequence. The Rsm22 dimer atomic model was generated manually by superposing two copies of a monomeric atomic model on the dimer *ab initio* shape. The fitting of the atomic models to the corresponding experimental SAXS scattering was calculated by *FoXS* (https://modbase.compbio.ucsf.edu/foxs/).

### 2.11. *S*-Adenosyl-l-methionine-dependent methyltransferase assay   

An *in vitro* methyltransferase assay was carried out using a modification of the protocol of Lee *et al.* (2007 ▸). Yeast mt-tRNAs were *in vitro* transcribed and purified for this experiment (see Sections S1–S6). Purified synthetic tRNAs were heated for 5 min at 70°C and cooled slowly to room temperature. 100 µl 0.5 m*M* *S*-adenosyl-l-(methyl-^3^H)-methionine stock solution was prepared by adding 2.5 µl 20 m*M* SAM, 10 µl 50 m*M* H_2_SO_4_ and 30 µl *S*-adenosyl-l-(methyl-^3^H)-methionine (320 Ci mol^−1^). Reactions were assembled on ice in 1.5 ml microcentrifuge tubes in a total volume of 30 µl. The reaction consisted of 20 µg monomeric Sc-Rsm22, varying amounts of tRNA depending on the experiment, 0.1 m*M* *S*-adenosyl-l-(methyl-^3^H)-methionine [specific activity 5.5 × 10^8^  disintegrations per minute (dpm) per micromole] and reaction buffer (100 m*M* Tris pH 8.0, 5 m*M* MgCl_2_, 100 m*M* KCl, 1 m*M* DTT, 0.01% Igepal CA-630). Three negative controls were used (the methylation reaction without Sc-Rsm22 enzyme, the methylation reaction without mt-tRNA and the methylation reaction with RNase-treated mt-tRNA). The reaction was carried out at 37°C for different time lengths depending on the experiment (see Section 3). After completion of the reaction time, 25 µl sample was taken from each reaction and precipitated with 380 µl ice-cold 50% trichloroacetic acid in a fresh tube for 20 min. A 5 µl aliquot was also removed to determine the total/input count of the label. The precipitated tRNA was centrifuged in a cold rotor (4°C) at 16 000*g* for 15 min. The pellet was then washed with 500 µl 50% trichloroacetic acid, followed by centrifugation at 16 000*g* for 15 min. The pellet was then dissolved in 100 µl 10 m*M* NaOH and transferred into scintillation plastic vials. 0.5 ml HiSafe solution was then added. Radioactivity was detected and measured in dpm from the reaction mixtures using a scintillation-counter detection device (Perkin Elmer TriCarb-2900TR liquid-scintillation analyzer). The time taken to generate data for each reaction mixture was 10 min.

Additionally, the activity of dimeric Sc-Rsm22 was also tested using a similar method as for monomeric Sc-Rsm22.

## 3. Results   

### 3.1. Bioinformatic characterization of Sc-Rsm22   

Sc-Rsm22 is a 628-amino-acid protein with a molecular mass of 72.2 kDa. The protein is highly alkaline, exhibiting a calculated pI of 9.9. Our bioinformatic analysis reveals that Sc-Rsm22 shows 18% sequence identity to Tb-Rsm22 and 16.5% to Mm-METTL17, and it can be concluded that these three enzyme homologues form an Rsm22 protein subfamily in the class I SAM-MTase family of proteins with a central SAM-binding core fold (Figs. 1 ▸ *a* and 1 ▸ *b*). We undertook detailed bioinformatic studies using various *in silico* tools. Carrying out comparative analyses employing several SAM-MTase family member homologues, we have generated an Sc-Rsm22 model and identified the key secondary-structure elements of the SAM-MT fold (Figs. 1 ▸ *a* and 1 ▸ *b*). The SAM-MTase fold is likely to start at position Met126 (the proposed starting position of α-helix αZ) and end at residue Asp462 (the end of β-strand β7) (Fig. 1 ▸ *b*). The conserved glycine-rich region at the end of β-strand β1 has the sequence Val-**Gly168**-Tyr-**Gly170**-Pro-**Ala172** in Sc-Rsm22, so the last glycine of the Gly-*X*-Gly-*X*-Gly motif is replaced by an alanine in this protein. Between β-strands β3 and β4 and between α-helix αE and β-strand β6, the Sc-Rsm22 protein also has several amino-acid sequence extensions exceeding 30 amino acids, which are very likely to be loop regions and are not present in other Rsm22-family members (Fig. 1 ▸ *b*). In contrast, Tb-Rsm22 has a long extension after α-helix αA which is not seen in the sequences of the Sc-Rsm22 and METTL17 proteins (Fig. 1 ▸ *b*).

The unique feature of the Rsm22 MTases is the conserved cysteine cluster comprising a zinc-finger loop structure at the end of the MTase fold. The cryo-EM structure of Tb-Rsm22 shows that this zinc-finger-like loop structure is embedded in the core β-sheet of the MTase fold, making a loop extension in the middle of β-strand β6. At the beginning of this loop structure, two cysteines and one histidine in the highly conserved sequence motif *X*-Ala-Pro-**Cys**-**His**-*X*-*X*-**Cys**-Pro-*X* coordinate the zinc ion together with a conserved cysteine at the end of the zinc-finger loop structure (Saurer *et al.*, 2019 ▸). In the Sc-Rsm22 sequence, these conserved zinc-binding residues are Cys373, His375, Cys379 and Cys400 (Fig. 1 ▸ *b*). In Tb-Rsm22 a fourth cysteine (Cys837) from the antiparallel β-sheet structure of the C-terminal domain also coordinates the bound zinc (Saurer *et al.*, 2019 ▸).

The C-terminal domain of Sc-Rsm22 (Asp462–Lys628) is predicted to contain both α-helices and β-strands (Fig. 1 ▸ *b*). It is strikingly shorter than the C-terminal domain of Tb-Rsm22 (which is 164 residues longer), but is 77 and 98 residues longer than those of Mm-METTL17 and Hs-METTL17, respectively. Because of these large differences in the lengths of the C-terminal regions, the standard sequence-alignment algorithm was unable to detect any significant sequence conservation. However, more careful interpretation of the sequences and comparison of the predicted secondary-structure elements of Sc-Rsm22 with the known structure of Tb-Rsm22 revealed a notable sequence similarity in the region Thr490–Lys530 corresponding to the region Ala815–Asp855 in Tb-Rsm22 (Fig. 1 ▸ *b*). This region interacts closely with the rRNA in the *T. brucei* mitoribosome complex, and the topology of this β-sheet structure found in Tb-Rsm22 strikingly resembles the so-called OB-fold, which is a structural motif that is frequently used for nucleic acid recognition (Theobald *et al.*, 2003 ▸). The OB-fold consist of two three-stranded antiparallel β-sheets and two α-helices with the topology α1–β1–β2–β3–α3–β4–β5. In the Tb-Rsm22 protein, the β1–β2–β3–α3–β4 region (Tyr819–Ile879) follows the OB-fold topology well when compared with the corresponding domain of the yeast aspartyl-tRNA synthase (AspRS) crystal structure (Ruff *et al.*, 1991 ▸), whereas α1 is completely missing and the last two β-strands (Gly882–Lys896) are slightly differently oriented. The first three β-strands of the OB-fold were also predicted to be present in the Sc-Rsm22 structure, as well as the α2 element (residues Gln531–Lys540), but the last two β-strands were not found. Cys837, which is found in the zinc-binding cysteine cluster in the Tb-Rsm22 structure, sits in the β3 strand of the OB-fold and is fully conserved in the Rsm22 protein family (Fig. 1 ▸ *b*). The secondary-structure prediction suggests that the OB-fold is followed by three C-terminal α-helices, and the first two helices form a coiled-coil motif according to *COILS* (Lupas *et al.*, 1991 ▸). This region does not share any noticeable sequence similarity with Sc-Rsm22. Also, no coiled-coil motif could be identified in Tb-Rsm22. The METTL17 proteins completely lack this C-terminal α-helical region according to our sequence analysis (Fig. 1 ▸ *b*).

The N-terminal region of Sc-Rsm22 (residues 1–125) is also predicted to be α-helical in our bioinformatic studies (Fig. 1 ▸ *b*). It is much shorter compared with that of Tb-Rsm22, with a length of 250 residues, but is around the same size in METTL17 proteins (Fig. 1 ▸ *b*). Interestingly, the mitochondrial isoform of Hs-METTL17 is completely devoid of the N-terminal domain (Fig. 1 ▸ *a*). Also, the sequence similarity is very limited within these three distant homologues. The N-terminus of Sc-Rsm22 shows the highest sequence similarity (33% with only 17% confidence) to the N-terminal region of the TrmB protein, which is a sugar-sensing transcriptional regulator from *Pyrococcus furiosus* (Krug *et al.*, 2006 ▸). In addition, some limited conservation was found with a few proteins of yeast lineage (the mitochondrial ribosomal protein of the small subunit from *Pichia stipites*, *Pichia pastoris* and *Candida dubliniensis*). The structures and functions of these proteins have not been characterized.

### 3.2. Production and purification of Sc-Rsm22   

**Figure 2:**
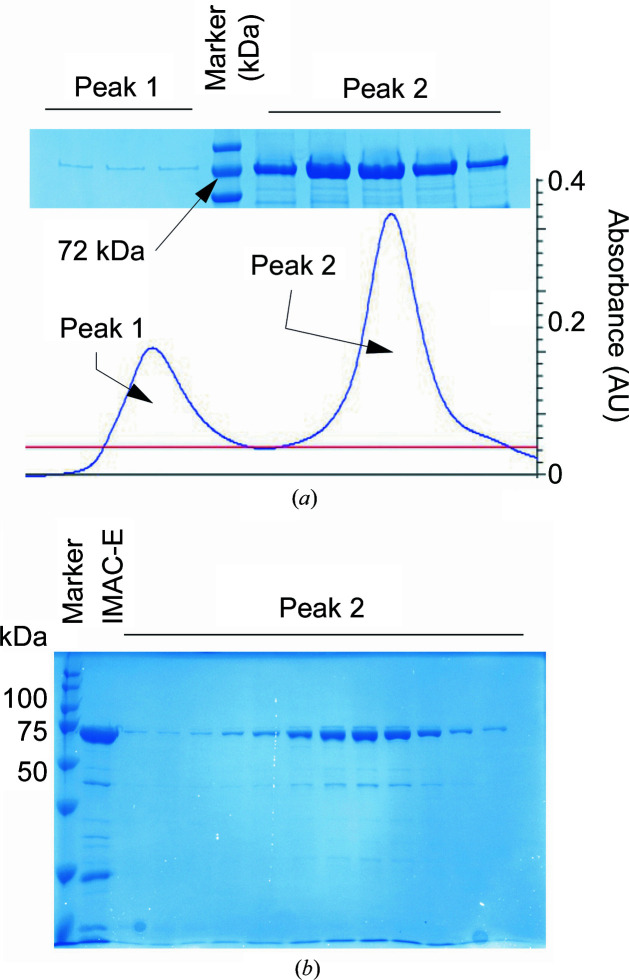
Purification of Sc-Rsm22. (*a*) Typical SEC elution profile of Sc-Rsm22 showing the presence of two oligomeric forms in the sample. The first peak (minor) and the second peak (major) correspond to higher (dimeric) and lower (monomeric) oligomeric forms of Sc-Rsm22, respectively. SDS–PAGE analysis of Sc-Rsm22 SEC elution fractions is shown at the top. (*b*) SDS–PAGE analysis of the SEC elution fractions of monomeric Sc-Rsm22. The samples in the SDS–PAGE were the following: molecular-mass standard marker, IMAC elution fraction (IMAC-E) and the SEC elution fractions of monomeric Sc-Rsm22 (peak 2). The bands of purified protein run just below the 75 kDa marker band, which corresponds to a calculated mass of 71.2 kDa for the histidine-tagged Sc-Rsm22 construct used in this study.

C-terminally His_6_-tagged Sc-Rsm22 (UniProt ID P36056; construct P36056; MA-S17–K628-HHHHHH) was expressed in *E. coli* by autoinduction as described in Section 2. 12 mg protein was obtained from 10 g harvested cells from 4 l culture. We set out to purify the protein to heterogeneity using IMAC and SEC. Analysis of the SEC fractions revealed peaks corresponding to two different oligomeric forms (Fig. 2 ▸ *a*). The higher oligomeric form of Sc-Rsm22 eluted at a volume of 10 ml (Fig. 2 ▸ *a*), whereas the lower oligomeric Sc-Rsm22 variant eluted at a volume of 15 ml, from a 24 ml SEC column. The purified protein fractions were analyzed by SDS–PAGE (Figs. 2 ▸ *a* and 2 ▸ *b*), and further with MS, which showed that the major band on SDS–PAGE (around 72 kDa) corresponded to Sc-Rsm22 carrying the C-terminal His tag. The additional band which was still present in the sample after the SEC step (Fig. 2 ▸ *b*) was identified to be a degradation product of Sc-Rsm22. Attempts at further purification using ion-exchange chromatography were unsuccessful (see Section 2).

### 3.3. Biophysical analyses of purified full-length Sc-Rsm22 by multi-angle light scattering and circular dichroism   

To assess the homogeneity of the protein preparations, both oligomeric forms of Sc-Rsm22 were individually subjected to SEC-MALS experiments (Supplementary Fig. S1). The variant with the longer elution time was confirmed to be a monomer with a determined molecular weight (MW) of 72 kDa, whereas the MALS MW of the other variant corresponded to a dimeric protein (MW of 152 kDa) (Supplementary Fig. S1*c*). Both peaks were homogeneous according to the MW distribution pattern (Supplementary Fig. S1*c*). The light-scattering signal revealed that both samples also contained aggregated protein, as well as a soluble protein peak with a MW of around 247 kDa. However, these peaks were not visible in the UV curves (Supplementary Figs. S1*a* and S1*b*), indicating that the percentages of these variants in the protein samples were very low.

**Figure 3:**
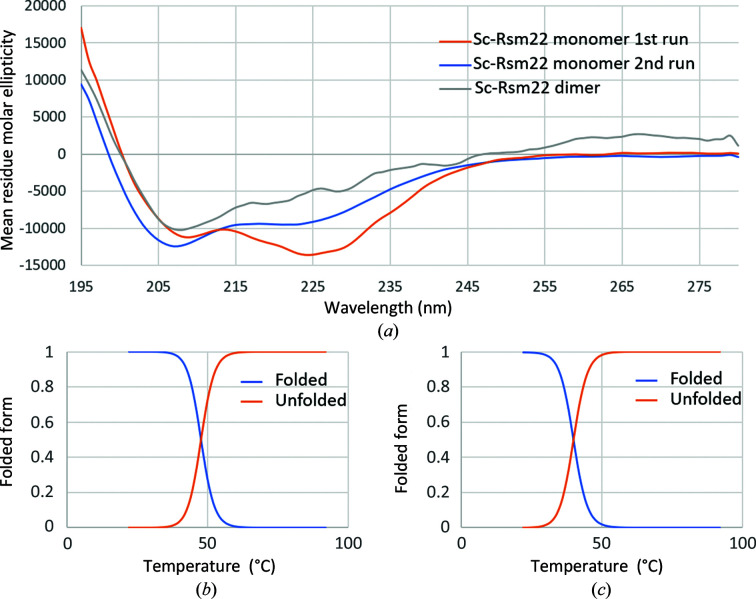
CD and thermostability analysis of Sc-Rsm22. (*a*) CD spectra of the Sc-Rsm22 monomer and dimer. The CD spectra in the wavelength range 250–270 nm indicate that monomeric Sc-Rsm22 has more α-­helical content than dimeric Sc-Rsm22 in 2.28 m*M* Tris pH 7.5, 28.5 m*M* NaCl, 0.28% glycerol. However, monomeric Sc-Rsm22 changes its structural fold in the direction of that of dimeric Sc-Rsm22 in the CD measurement solvent. The change in fold is rapid at the beginning of the solvent exchange from higher to lower salt concentration but stabilizes within 30 min. The figure shows the CD spectra of the Sc-Rsm22 monomer collected just after changing the solvent (first run, orange) and after 30 min (second run, blue) and of the Sc-Rsm22 dimer (gray). (*b*) The melting curve of dimeric Sc-Rsm22 shows a *T*_m_ value of 48°C. (*c*) The melting curve of the Sc-Rsm22 monomer shows a *T*_m_ value of 39°C.

Both variants of the full-length Sc-Rsm22 were well folded according to CD analysis (Fig. 3 ▸ *a*). The spectra of the monomeric form showed a somewhat higher calculated helical content (34.9%) compared with the dimeric form, where the calculated helical content was 27.2%. The dimeric form was also more stable than the monomeric form; the thermal stability (*T*_m_) values were 48 and 39°C, respectively (Figs. 3 ▸ *b* and 3 ▸ *c*). Raw data of thermal unfolding (data from 80 to 92°C are not shown as they are not significant) are presented in Supplementary Fig. S3. We observed a change in the structural fold of the Sc-Rsm22 monomer in solvent containing a lower salt concentration (28.5 m*M* NaCl) from that present in the purification solvent. This change was rapid at the beginning of the solvent change from higher salt concentration (500 m*M* NaCl) to lower salt concentration and stabilized within 30 min. We initially collected CD spectra using monomeric Sc-Rsm22 for three repetitions, but did not generate an average of these repetitions as the data deviated from each other. We used the first set of data for CD analysis (Fig. 3 ▸ *a*). In addition, a second measurement of CD spectra after 30 min showed that the change was directing Sc-Rsm22 dimer formation from Sc-Rsm22 monomers (Supplementary Table S4). We collected CD spectra with ten repetitions after 30 min of dilution and observed that the protein became stable (Supplementary Fig. S2*b*). A second CD analysis of the Sc-Rsm22 monomer was also performed using the last set of data (Supplementary Table S4). However, unlike those of the Sc-Rsm22 monomer, the CD spectra of the Sc-Rsm22 dimer were stable at low salt concentration (Supplementary Fig. S2*a*). The CD spectra of the Sc-Rsm22 dimer shown in Fig. 3 ▸(*a*) (Sc-Rsm22 dimer in gray) are the average of three independent data sets.

### 3.4. Small-angle X-ray scattering (SAXS) structure of Sc-Rsm22   

**Figure 4:**
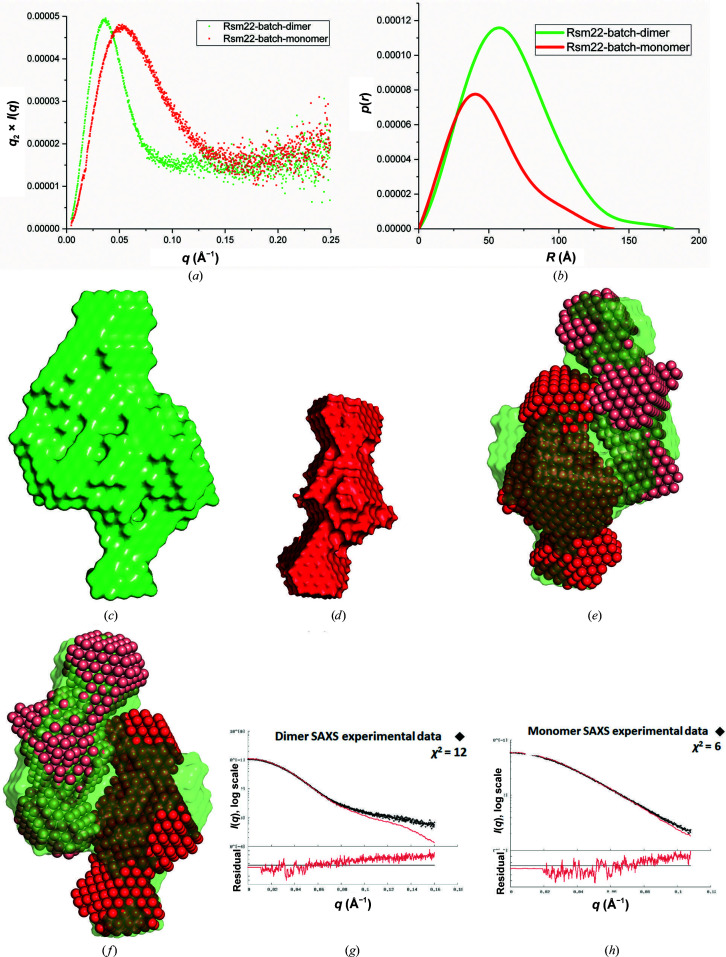
SAXS analysis of Sc-Rsm22. (*a*) Kratky scattering of both the Sc-Rsm22 monomer and dimer shows that the proteins are folded. (*b*) Pair distribution curve of both the Sc-Rsm22 monomer and dimer, showing *D*_max_ values of 181 and 138 Å for the dimer and monomer, respectively. (*c*) The *ab initio* shape of the Sc-Rsm22 dimer. (*d*) The *ab initio* shape of the Sc-Rsm22 monomer. (*e*) Two identical Sc-Rsm22 monomeric *ab initio* shapes (red and salmon) are superimposed with the *ab initio* model of dimeric Sc-Rsm22 (green). (*f*) A 180° rotation of the superimposed model shown in (*e*). (*g*) A fitting curve of the Sc-Rsm22 dimer *ab initio* model against the experimental SAXS data calculated by the *FoXS* server. (*h*) A fitting curve of the Sc-Rsm22 monomer *ab initio* model against the experimental SAXS data calculated by the *FoXS* server.

Sc-Rsm22 exhibits two distinct forms under the conditions that we investigated. We performed SAXS studies on the dimeric and monomeric variants in batch mode. The *ab initio* shapes generated for both forms and SAXS analysis are shown in Fig. 4 ▸. Kratky scattering analysis of the Sc-Rsm22 monomer and dimer suggested that both protein variants were folded (Fig. 4 ▸ *a*). The pair distribution curves of the Sc-Rsm22 monomer and dimer suggested *D*_max_ values of 138 and 181 Å for the monomer and dimer, respectively (Fig. 4 ▸ *b*). The *ab initio* shapes of the Sc-Rsm22 dimer and monomer are shown in Figs. 4 ▸(*c*) and 4 ▸(*d*), respectively. We estimated the Porod volume for the monomer as 160 000 Å^−3^ (data used 1–367). In this representation, the monomeric protein displays an elongated shape with a large globular domain in the center and two smaller globular bulges at each end (Fig. 4 ▸ *d*). The dimeric form, with a Porod volume of 521 000 Å^−3^ (data used 1–290), appears as an antiparallel association of the monomers, with a larger central bulge and smaller bulges at each end of the structure. Unlike the MALS data, the molecular mass determined for the dimer appears to be exaggerated in the SAXS analysis (Table 1 ▸). However, superimposition of the two Sc-Rsm22 monomeric *ab initio* shapes on the Sc-Rsm22 dimer *ab initio* shape showed a very good match (Fig. 4 ▸ *e*). The dimer is depicted by a green surface, while the two monomers are shown as red and salmon surfaces. We also carried out SEC-SAXS (online SAXS) experiments. The parameters calculated for monomeric Sc-Rsm22 from both the batch and online experiments are nearly the same (Table 1 ▸). However, dimeric Sc-Rsm22 showed a larger Porod volume (638 000 Å^−3^) in online experiments compared with batch experiments. Like the batch-mode experiments, superimposition of the *ab initio* models of two copies of monomeric Sc-Rsm22 on one copy of dimeric Sc-Rsm22 shows a good fit (Supplementary Fig. S4). The SAXS parameters calculated for the Sc-Rsm22 dimer and monomer from both batch and online experiments are provided in Table 1 ▸.

### 3.5. *In vitro* methyltransferase assay/SAM-dependent methyltransferase assay   

A SAM [*S*-adenosyl-l-(methyl-^3^H)-methionine]-dependent methyltransferase assay was performed *in vitro* to test the methyltransferase activity of Sc-Rsm22. Because yeast mitochondrial tRNAs are heavily methylated, whereas the rRNAs are not, we only concentrated on studying the methylation of various tRNA molecules in this study. We used 38.8 pmol of different mt-tRNAs as substrates for Sc-Rsm22, with a mixture of radioactively labeled and cold SAM as the methyl-group donor in the reactions. The amount of methyl groups transferred to the substrates was determined by measuring the radioactivity of the samples with a scintillation counter (Perkin Elmer TriCarb-2900TR liquid scintillation analyzer) after completion of the reaction followed by trichloro­acetic acid precipitation. The radioactivity was measured in the form of disintegrations per minute (dpm). A total of 18 Mt-tRNAs were tested as substrates and it was found that all of them incorporated the radioactive label from the substrate in the presence of monomeric Sc-Rsm22. However, we were unable to detect any activity for the dimeric variant of Sc-Rsm22. The obtained dpm values for different mt-tRNAs, indicating the incorporation of tritiated methyl groups, are presented in Supplementary Table S7. The data show that the values are between 500 and 850 dpm on average after subtracting the negative control value from each sample, with no clear indication of tRNA substrate preference (Supplementary Table S7). Data for a minimum of three and a maximum of four experiments were obtained for each tRNA.

**Figure 5:**
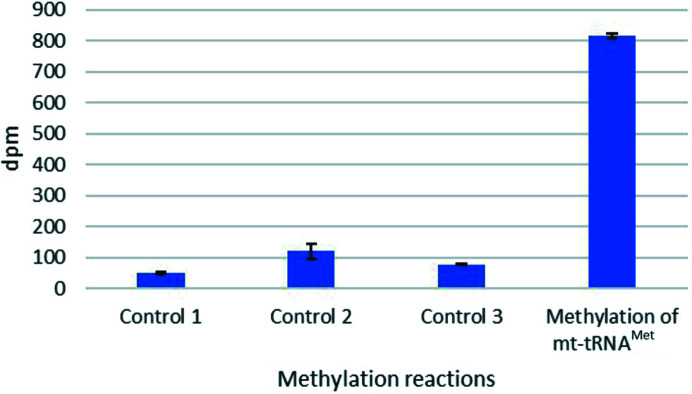
Example experiment for the methylation of mt-tRNA^Met^ by monomeric Sc-Rsm22. In controls 1–3, either the enzyme Sc-Rsm22 was omitted (control 1), mt-tRNA^Met^ was omitted (control 2) or the reaction mixture was treated with RNase (control 3). All three controls are missing at least one essential component for the reaction, preventing a specific methylation reaction. The measured dpm values for the different negative controls were in the range 47–145. The average dpm value for mt-tRNA^Met^ methylation is 816.

Fig. 5 ▸ illustrates these measurements using mt-tRNA^Met^ as an example for the enzymatic assays of tRNA methylation *in vitro* that we carried out. We included multiple negative control reactions to ensure the specificity of Rsm22-dependent incorporation of methyl group(s) into this tRNA. The control reaction was carried out without enzyme and with RNase-treated sample. It is seen that the dpm values for the different negative controls range between 47 and 145.

In addition, the methyltransferase activity of Sc-Rsm22 using mt-tRNA^Met^ as a substrate was studied by varying the amount of substrate and the reaction time. The substrate concentration-dependent methyltransferase activities measured for mt-tRNA^Met^ are shown in Supplementary Fig. S5(*a*). The methyl-group incorporation increased linearly with the increase in substrate (mt-tRNA^Met^) in the range 19.3–57.96 pmol. The *R* ^2^ value is 0.93. For the reaction time-dependent experiments, the concentrations of all of the reagents, including substrates (32.2 pmol mt-tRNA^Met^), and enzyme were unchanged. We collected data at different time points (5, 10, 15, 20, 30 and 40 min) and the incorporation of methyl groups increased with time up to the 30 min mark, after which the reaction appeared to reach a steady state. This indicates that the reaction becomes saturated after about 30 min (Supplementary Fig. S5*b*) and is consistent with an enzyme-catalyzed reaction.

## 4. Discussion   

Here, we report the expression and purification of Sc-Rsm22 in *E. coli*, determine a solution structure of the protein and demonstrate its enzymatic function. Sc-Rsm22 is a conserved nuclear-encoded mitochondria-localized protein in *S. cerevisiae* that has been shown to physically interact with the small subunit of the mitochondrial ribosome (Saveanu *et al.*, 2001 ▸). We previously reported that mutations in *RSM22* act as synthetic ‘petites’ in conjunction with a diminished function of enoyl reductase variant operating in the mtFAS process (Kursu *et al.*, 2013 ▸). MtFAS has been implicated in the post-translational control of mitochondrial gene expression in *S. cerevisiae*, and it is possible that the Sc-Rsm22 protein plays a role in mediating translational control in mitochondria.

**Figure 6:**
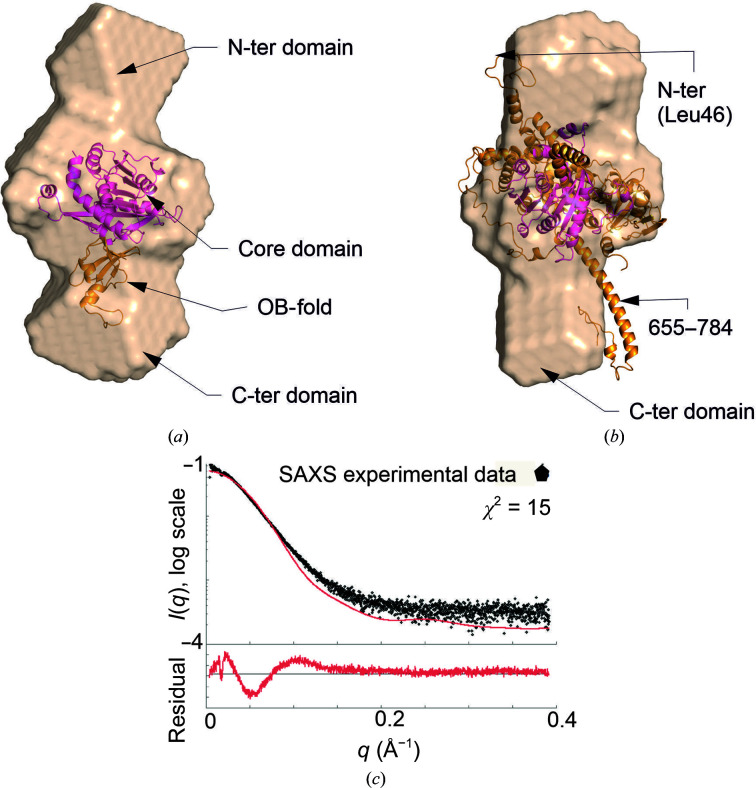
Three-domain structure of Sc-Rsm22. (*a*) The *ab initio* dummy-atom model of monomeric Sc-Rsm22 aligned with the truncated Tb-Rsm22 cryo-EM structure including the SAM-MTase domain (extended loop regions have been removed) and the OB-motif of the C-terminal domain. The zinc-finger motif of the SAM-MTase domain, corresponding to the region Lys126–Lys461 in Sc-Rsm22, is included in this structural model of Sc-Rsm22. The hypothesis is that the SAM-MTase core fold is located in the middle of the elongated solution structure extended by the unknown N-terminal and potential OB-fold C-terminal structures. (*b*) The *ab initio* dummy-atom model of monomeric Sc-Rsm22 aligned with full-length Tb-Rsm22. The N- and C-termini of the Tb-Rsm22 cryo-EM structure, as well as part of the extended loop regions (655–784) (see Fig. 1 ▸ *b*), are highlighted in (*b*). (*c*) A fitting curve of the full-length Tb-Rsm22 coordinates [the same as used in (*b*)] against the experimental SAXS data.

A recently published cryo-EM structure of the *T. brucei* mitochondrial ribosome revealed the structural fold and interactions of Tb-Rsm22 (Saurer *et al.*, 2019 ▸). Because this enzyme is the closest homologue of Sc-Rsm22, it allowed us to generate the first structural model of Sc-Rsm22 together with experimentally calculated SAXS data. The SAM-MTase core domain is expected to be located in the middle of the elongated Sc-Rsm22 solution structure (Fig. 6 ▸ *a*). The MTase domain in Sc-Rsm22 is compact according to the SAXS structure and does not contain any elongated loop regions as seen in the Tb-Rsm22 structure (Figs. 1 ▸ *b* and 6 ▸). Tb-Rsm22 harbors extended helical regions before α-helix αZ and after α-helix αA, which mainly interact with other methyltransferases in the *T. brucei* mitoribosome complex. In contrast, Sc-Rsm22 includes smaller extensions in its sequence after β-strand β3 and α-helix αE which are not present in Tb-Rsm22. Interestingly, METTL17 enzymes lack all four of the extensions found in Tb-Rsm22 or Sc-Rsm22 (Fig. 1 ▸ *b*). In general, SAM-MTase structures bind their substrate with the loops between β4 and αD, β5 and αE, and β6 and β7 (Martin & McMillan, 2002 ▸), and this can also be seen in RNA methyltransferases such as Trm5 in complex with tRNA^Cys^ (Goto-Ito *et al.*, 2009 ▸) and RumA in complex with rRNA (Lee *et al.*, 2005 ▸). In the Rsm22 proteins, the two first loop regions have roughly the same length, but the third loop region is more extended in Sc-Rsm22. The first two loops do not contain any conserved positively charged residues, as would be expected for nucleic acid binding and as seen in Trm5 and RumA, but the extended loop of Sc-Rsm22 contains four arginines and one lysine (Arg434, Lys436, Arg440, Arg441 and Arg444), which may play a role in RNA binding (Fig. 1 ▸ *b*). Interestingly, this region does not correspond to the portion of Tb-Rsm22 interacting with the rRNA in the *T. brucei* mito­ribosome complex (Saurer *et al.*, 2019 ▸). Instead, the positively charged residues (Lys607-Arg608-Lys609-Arg610) in the unique zinc-finger structure are heavily involved in rRNA binding. This Lys/Arg-rich sequence quartet is not conserved in the other Rsm22 proteins, but Sc-Rsm22 also contains three lysine residues (Lys392, Lys395 and Lys398) in this region. However, the role of the zinc-finger structure in RNA binding is not evident in Sc-Rsm22 or in the METTL17 proteins.

The OB-domain is a commonly found fold in proteins that interact with nucleic acids (Theobald *et al.*, 2003 ▸). We noticed that this domain is also present in Tb-Rsm22 and very likely also in other Rsm22-family members, including Sc-Rsm22. This domain is located after the SAM-MTase fold in the sequence and these two domains closely interact via the conserved cysteine of the OB-fold, which also interacts with the zinc bound to the SAM-MTase zinc-finger motif. According to our bioinformatic characterization, the OB-fold may not be complete in Sc-Rsm22, but contains at least the three first β-strands and the second α-helix (*i.e.* the β1–β2–β3–α3 motif) of this fold, including residues Thr490–Gly542. The corresponding motif is highly similar in all Rsm22-family members (Fig. 1 ▸ *b*) and makes several protein–rRNA interactions in the Tb-Rsm22 structure. In Sc-Rsm22, this region includes ten positively charged residues, which are potential candidates for RNA binding. The most likely candidates for such an interaction are Lys503, Arg504, Lys505, Lys521, Arg527, Arg538 and Lys539. AspRS also interacts with the tRNA molecule (with the anticodon bases) via the OB-domain (Ruff *et al.*, 1991 ▸). We compared the binding interactions of the OB-folds of Tb-Rsm22 and AspRS and noticed that they were very similar, particularly within the β1–β2–β3–α3 motif. One of the key regions for the RNA interaction is the β2–β3 loop of the OB-fold, with one conserved glycine at the tip of the loop. This glycine is also conserved in Sc-Rsm22 but not in METTL17 proteins, and corresponds to Gly506 (Fig. 1 ▸ *b*). It is noteworthy that Glu520, a previously reported point mutation (Glu520Gly) of which in Sc-Rsm22 in combination with compromised mtFAS causes a respiratory deficiency phenotype in yeast (Kursu *et al.*, 2013 ▸), is located in the β3 strand of the OB-fold. According to our model structure of Sc-Rsm22, Glu520 does not directly interact with the RNA, but it is very likely that the neighboring Lys521 does. Therefore, the Glu520Gly mutation may change the folding of the OB-fold and disrupt the RNA-binding properties of Sc-Rsm22.

Our solution structure of Sc-Rsm22 also proposes that the yeast protein is very different compared with the Tb-Rsm22 structure characterized as part of the *T. brucei* mitoribosome (Saurer *et al.*, 2019 ▸). This is expected because the N- and C-terminal regions of these two Rsm22 family members share very limited sequence homology and both enzymes have several long extensions in the sequence which are not present in the other family members. This strongly suggests that these two enzymes methylate different molecules and and/or have different physiological functions. Sc-Rsm22 may associate similarly with the mitoribosome as Tb-Rsm22, as also shown earlier (Saveanu *et al.*, 2001 ▸), but the interaction must be less tight, as Sc-Rsm22 was not present in the cryo-EM structure of the yeast mitoribosome (Desai *et al.*, 2017 ▸). We cannot conclude from our bioinformatics data which region(s) of Sc-Rsm22 could take part in ribosomal interactions and whether the regions are the same as those used for binding tRNA, which we propose to be the substrate of Sc-Rsm22. The zinc-finger motif of the MTase domain and the preceding OB-domain are both very likely to play a role in RNA binding in Sc-Rsm22. Tb-Rsm22 interacts with rRNA using both of these motifs, but this may not be true for Sc-Rsm22, which is expected to associate more loosely with the mitoribosome. Sc-Rsm22 may use the OB-fold for tRNA binding, as seen for example in AspRS (Ruff *et al.*, 1991 ▸), but confirmation or rejection of this suggestion this requires further studies.

The recombinantly produced Sc-Rsm22 forms both monomers and dimers, which it was possible to separate by SEC (Fig. 2 ▸) and characterize employing SAXS (Fig. 4 ▸), MALS (Supplementary Fig. S1), DLS and CD (Fig. 3 ▸ and Supplementary Table S4) techniques. Our bioinformatic analyses predicted 232 residues to be present in helical structure in Sc-Rsm22, which is 37% of the total residues. Monomeric Sc-Rsm22 showed a similar helical content in CD analyses. A question of the physiological form of Sc-Rsm22 thus arises. The data presented here, including our functional analyses, indicate that the monomeric variant is likely to be the physiologically relevant form of the protein. However, we cannot completely exclude the possibility of an additional physiological function of the dimeric variant of Sc-Rsm22, as MALS and SAXS analyses as well as thermal unfolding data suggest the formation of dimeric Sc-Rsm22.

In addition to the structural and biophysical analyses, our functional analyses of Sc-Rsm22 also establish its function as an RNA-methylating enzyme. Previous studies have already concluded that Sc-Rsm22 is likely to be a SAM-dependent methyltransferase, and both proteins and RNA molecules have been proposed to be substrates of this enzyme (Wlodarski *et al.*, 2011 ▸; Szczepińska *et al.*, 2014 ▸). Our data clearly indicate that Sc-Rsm22 mediates the transfer of tritiated methyl groups from radioactively labeled SAM to a variety of yeast mt-tRNAs. We were, however, unable to determine a specificity for any particular tRNA variant. This may also be the case *in vivo*, but may simply reflect that we have measured enzymatic activity removed from the small ribosomal subunit context. The question of the physiological RNA substrate is therefore not completely settled. We do not favor the idea of rRNA as the substrate, because previous studies demonstrated that Sc-Rsm22 is a small mitoribosomal subunit, and small ribosomal subunit rRNAs in yeast mitochondria are not methylated (Klootwijk *et al.*, 1975 ▸). In contrast, mt-tRNAs are heavily modified at least at 90 different positions. Many of these modifications are methylations, and only a few tRNA-methylating enzymes have been characterized to date (Machnicka *et al.*, 2014 ▸). Our bioinformatic, structural and enzymatic analyses provided clues to the potential substrates of Sc-Rsm22. Considering all information, Sc-Rsm22 is likely to be a tRNA methyltransferase.

In conclusion, our studies have revealed several important features of the Rsm22 family of SAM-dependent methyltransferases. The Sc-Rsm22 enzyme is capable of the transfer of methyl groups from SAM to mt-tRNAs. We propose that the methylation of mt-tRNAs by Sc-Rsm22 is needed for mitochondrial respiration in yeast. Our careful bioinformatic analysis with multiple sequence-alignment studies shows that Sc-Rsm22 has a three-domain composition consisting of (i) an N-terminal α-helical domain with unknown function, (ii) a central MTase core responsible for the methyltransferase activity and (iii) a C-terminal domain including an OB-fold that is responsible for binding the RNA substrate, probably tRNA, extended by an α-helical extension which may form a coiled-coil structure. However, many open questions still remain concerning the structure and function of this mitochondrial enzyme. Crystallographic studies have been initiated to solve the high-resolution crystal structure of this enzyme.

## Supplementary Material

SASBDB reference: Sc-Rsm22 monomer, SASDJS5

SASBDB reference: Sc-Rsm22 dimer, SASDJT5

Information on oligonucleotides and PCR conditions; Supplementary Tables and Figures. DOI: 10.1107/S2059798321004149/jc5035sup1.pdf

## Figures and Tables

**Table 1:** Parameters used for SAXS data collection and analysis –, not applicable.

Parameters	Sc-Rsm22 dimer (batch)	Sc-Rsm22 monomer (batch)	Sc-Rsm22 dimer (online)	Sc-Rsm22 monomer (online)
SEC-SAXS column	—	—	Superdex 200 5/150, GE Healthcare	Superdex 200 5/150, GE Healthcare
Solvent	40 m*M* Tris pH 7.5, 500 m*M* NaCl, 5% glycerol, 2.5 m*M* DTT	40 m*M* Tris pH 7.5, 500 m*M* NaCl, 5% glycerol, 2.5 m*M* DTT	40 m*M* Tris pH 7.5, 500 m*M* NaCl, 5% glycerol, 2.5 m*M* DTT	40 m*M* Tris pH 7.5, 500 m*M* NaCl, 5% glycerol, 2.5 m*M* DTT
Protein concentration (mg ml^−1^)	5	2.9	†	†
Injected volume (µl)	—	—	30	30
Flow rate (ml min^−1^)	—	—	0.15	0.15
Sample temperature (°C)	20	20	20	20
Beamline	B21, DLS	B21, DLS	BM29, ESRF	BM29, ESRF
Beam size (µm)	250 × 250	250 × 250	50 × 50	50 × 50
Detector	PILATUS 2M	PILATUS 2M	PILATUS 1M	PILATUS 1M
Wavelength (Å)	1	1	1	1
SAXS data reduction	*I*(*q*) versus *q*, solvent subtraction using *SCÅTTER*	*I*(*q*) versus *q*, solvent subtraction using *SCÅTTER*	*I*(*q*) versus *q*, solvent subtraction using *SCÅTTER*	*I*(*q*) versus *q*, solvent subtraction using *SCÅTTER*
Basic analysis: Guiner, *p*(*r*)	*PRIMUS* from *ATSAS*, *SCÅTTER*	*PRIMUS* from *ATSAS*, *SCÅTTER*	*PRIMUS* from *ATSAS*, *SCÅTTER*	*PRIMUS* from *ATSAS*, *SCÅTTER*
*I*(0) (cm^−1^)	0.1	0.06	3.17	3.85
*R*_g_ (Å)	50.4	39.6	53.2	38.5
*D*_max_ (Å)	182	139	168	128
Porod volume (Å^−3^)	521000	160000	638000	148000
Molecular weight (kDa)				
From MM_qp_	272	91	266	66
From MoW	201	81	297	81
From *V*_c_	234	82	—	78
From size and shape	287	111	314	99
Shape/bead modeling	*DAMMIN*, *ATSAS* online	*DAMMIN*, *ATSAS* online	*DAMMIN*, *ATSAS* online	*DAMMIN*, *ATSAS* online
3D graphic model representation	*PyMOL*	*PyMOL*	*PyMOL*	*PyMOL*
SASBDB code	SASDJT5	SASDJS5	—	—

† 2.85 mg ml^−1^ concentrated mixture of dimeric and monomeric ScRsm22 with unknown percentages.

## References

[bb1] Boczonadi, V., Ricci, G. & Horvath, R. (2018). *Essays Biochem.* **62**, 321–340. 10.1042/EBC20170103 PMC6056718 29980628

[bb2] Desai, N., Brown, A., Amunts, A. & Ramakrishnan, V. (2017). *Science*, **355**, 528–531. 10.1126/science.aal2415 PMC5295643 28154081

[bb3] Dimmer, K. S., Fritz, S., Fuchs, F., Messerschmitt, M., Weinbach, N., Neupert, W. & Westermann, B. (2002). *Mol. Biol. Cell*, **13**, 847–853. 10.1091/mbc.01-12-0588 PMC99603 11907266

[bb4] Du, P., Yuan, B., Cao, J., Zhao, J., Ding, L., Chen, L., Ying, S., Jiang, L., Lin, J., Xu, X., Cheng, L. & Ye, Q. (2015). *IUBMB Life*, **67**, 861–868. 10.1002/iub.1444 26488768

[bb5] Emanuelsson, O., Brunak, S., von Heijne, G. & Nielsen, H. (2007). *Nat. Protoc.* **2**, 953–971. 10.1038/nprot.2007.131 17446895

[bb6] Emanuelsson, O., Nielsen, H., Brunak, S. & von Heijne, G. (2000). *J. Mol. Biol.* **300**, 1005–1016. 10.1006/jmbi.2000.3903 10891285

[bb7] Förster, S., Apostol, L. & Bras, W. (2010). *J. Appl. Cryst.* **43**, 639–646.

[bb8] Goto-Ito, S., Ito, T., Kuratani, M., Bessho, Y. & Yokoyama, S. (2009). *Nat. Struct. Mol. Biol.* **16**, 1109–1115. 10.1038/nsmb.1653 19749755

[bb9] Hajizadeh, N. R., Franke, D., Jeffries, C. M. & Svergun, D. I. (2018). *Sci. Rep.* **8**, 7204. 10.1038/s41598-018-25355-2 PMC5940760 29739979

[bb10] Hoffman, C. S. & Winston, F. (1987). *Gene*, **57**, 267–272. 10.1016/0378-1119(87)90131-4 3319781

[bb11] Johnston, I. G. & Williams, B. P. (2016). *Cell. Syst.* **2**, 101–111. 10.1016/j.cels.2016.01.013 27135164

[bb12] Kastaniotis, A. J., Autio, K. J., Sormunen, R. T. & Hiltunen, J. K. (2004). *Mol. Microbiol.* **53**, 1407–1421. 10.1111/j.1365-2958.2004.04191.x 15387819

[bb13] Kelley, L. A., Mezulis, S., Yates, C. M., Wass, M. N. & Sternberg, M. J. (2015). *Nat. Protoc.* **10**, 845–858. 10.1038/nprot.2015.053 PMC5298202 25950237

[bb14] Klootwijk, J., Klein, I. & Grivell, L. A. (1975). *J. Mol. Biol.* **97**, 337–350. 10.1016/s0022-2836(75)80044-1 1102710

[bb15] Konarev, P. V., Volkov, V. V., Sokolova, A. V., Koch, M. H. J. & Svergun, D. I. (2003). *J. Appl. Cryst.* **36**, 1277–1282.

[bb16] Kozin, M. B. & Svergun, D. I. (2001). *J. Appl. Cryst.* **34**, 33–41.

[bb17] Krug, M., Lee, S. J., Diederichs, K., Boos, W. & Welte, W. (2006). *J. Biol. Chem.* **281**, 10976–10982. 10.1074/jbc.M512809200 16473881

[bb18] Kursu, V. A., Pietikäinen, L. P., Fontanesi, F., Aaltonen, M. J., Suomi, F., Raghavan Nair, R., Schonauer, M. S., Dieckmann, C. L., Barrientos, A., Hiltunen, J. K. & Kastaniotis, A. J. (2013). *Mol. Microbiol.* **90**, 824–840. 10.1111/mmi.12402 PMC4153884 24102902

[bb19] Lee, C., Kramer, G., Graham, D. E. & Appling, D. R. (2007). *J. Biol. Chem.* **282**, 27744–27753. 10.1074/jbc.M704572200 17652090

[bb20] Lee, T. T., Agarwalla, S. & Stroud, R. M. (2005). *Cell*, **120**, 599–611. 10.1016/j.cell.2004.12.037 15766524

[bb21] Lupas, A., Van Dyke, M. & Stock, J. (1991). *Science*, **252**, 1162–1164. 10.1126/science.252.5009.1162 2031185

[bb22] Machnicka, M. A., Olchowik, A., Grosjean, H. & Bujnicki, J. M. (2014). *RNA Biol.* **11**, 1619–1629. 10.4161/15476286.2014.992273 PMC4615829 25611331

[bb23] Martin, J. L. & McMillan, F. M. (2002). *Curr. Opin. Struct. Biol.* **12**, 783–793. 10.1016/s0959-440x(02)00391-3 12504684

[bb24] McKenney, K. M., Rubio, M. A. T. & Alfonzo, J. D. (2017). *Enzymes*, **41**, 51–88. 10.1016/bs.enz.2017.03.002 PMC6589034 28601226

[bb25] Murphy, E., Ardehali, H., Balaban, R. S., DiLisa, F., Dorn, G. W., Kitsis, R. N., Otsu, K., Ping, P., Rizzuto, R., Sack, M. N., Wallace, D., Youle, R. J. & American Heart Association Council on Basic Cardiovascular Sciences, Council on Clinical Cardiology, and Council on Functional Genomics and Translational Biology (2016). *Circ. Res.* **118**, 1960–1991. 10.1161/RES.0000000000000104 PMC6398603 27126807

[bb26] Ruff, M., Krishnaswamy, S., Boeglin, M., Poterszman, A., Mitschler, A., Podjarny, A., Rees, B., Thierry, J. C. & Moras, D. (1991). *Science*, **252**, 1682–1689. 10.1126/science.2047877 2047877

[bb27] Saurer, M., Ramrath, D. J. F., Niemann, M., Calderaro, S., Prange, C., Mattei, S., Scaiola, A., Leitner, A., Bieri, P., Horn, E. K., Leibundgut, M., Boehringer, D., Schneider, A. & Ban, N. (2019). *Science*, **365**, 1144–1149. 10.1126/science.aaw5570 31515389

[bb28] Saveanu, C., Fromont-Racine, M., Harington, A., Ricard, F., Namane, A. & Jacquier, A. (2001). *J. Biol. Chem.* **276**, 15861–15867. 10.1074/jbc.M010864200 11278769

[bb29] Schubert, H. L., Blumenthal, R. M. & Cheng, X. (2003). *Trends Biochem. Sci.* **28**, 329–335. 10.1016/S0968-0004(03)00090-2 PMC2758044 12826405

[bb30] Sharma, M. R., Koc, E. C., Datta, P. P., Booth, T. M., Spremulli, L. L. & Agrawal, R. K. (2003). *Cell*, **115**, 97–108. 10.1016/s0092-8674(03)00762-1 14532006

[bb31] Shi, Z., Xu, S., Xing, S., Yao, K., Zhang, L., Xue, L., Zhou, P., Wang, M., Yan, G., Yang, P., Liu, J., Hu, Z. & Lan, F. (2019). *FASEB J.* **33**, 13040–13050. 10.1096/fj.201901331R 31487196

[bb32] Svergun, D. I. (1992). *J. Appl. Cryst.* **25**, 495–503.

[bb33] Svergun, D. I. (1999). *Biophys. J.* **76**, 2879–2886. 10.1016/S0006-3495(99)77443-6 PMC1300260 10354416

[bb34] Swinehart, W. E., Henderson, J. C. & Jackman, J. E. (2013). *RNA*, **19**, 1137–1146. 10.1261/rna.039651.113 PMC3708533 23793893

[bb35] Szczepińska, T., Kutner, J., Kopczyński, M., Pawłowski, K., Dziembowski, A., Kudlicki, A., Ginalski, K. & Rowicka, M. (2014). *PLoS Comput. Biol.* **10**, e1003514. 10.1371/journal.pcbi.1003514 PMC3961171 24651469

[bb36] Theobald, D. L., Mitton-Fry, R. M. & Wuttke, D. S. (2003). *Annu. Rev. Biophys. Biomol. Struct.* **32**, 115–133. 10.1146/annurev.biophys.32.110601.142506 PMC1564333 12598368

[bb37] Torres, A. G., Batlle, E. & Ribas de Pouplana, L. (2014). *Trends Mol. Med.* **20**, 306–314. 10.1016/j.molmed.2014.01.008 24581449

[bb38] Týč, J., Novotná, L., Peña-Diaz, P., Maslov, D. A. & Lukeš, J. (2017). *Mitochondrion*, **34**, 67–74. 10.1016/j.mito.2017.01.003 28089944

[bb39] Van Vranken, J. G., Nowinski, S. M., Clowers, K. J., Jeong, M. Y., Ouyang, Y., Berg, J. A., Gygi, J. P., Gygi, S. P., Winge, D. R. & Rutter, J. (2018). *Mol. Cell*, **71**, 567–580. 10.1016/j.molcel.2018.06.039 PMC6104058 30118679

[bb40] Wallace, D. C. (2007). *Annu. Rev. Biochem.* **76**, 781–821. 10.1146/annurev.biochem.76.081205.150955 17506638

[bb41] Waterhouse, A., Bertoni, M., Bienert, S., Studer, G., Tauriello, G., Gumienny, R., Heer, F. T., de Beer, T. A. P., Rempfer, C., Bordoli, L., Lepore, R. & Schwede, T. (2018). *Nucleic Acids Res.* **46**, W296–W303. 10.1093/nar/gky427 PMC6030848 29788355

[bb42] Wlodarski, T., Kutner, J., Towpik, J., Knizewski, L., Rychlewski, L., Kudlicki, A., Rowicka, M., Dziembowski, A. & Ginalski, K. (2011). *PLoS One*, **6**, e23168. 10.1371/journal.pone.0023168 PMC3153492 21858014

