# Supplementary material for: Expression and analysis of the SAM-dependent RNA methyltransferase Rsm22 from Saccharomyces cerevisiae
Source: Acta Crystallogr D Struct Biol. 2021 May 19;77(Pt 6):840–53. doi: 10.1107/S2059798321004149 (PMC8171064; doi:10.1107/S2059798321004149)
Supplement: Supplementary file 1 [file d-77-00840-sup1.pdf]

STRUCTURAL
BIOLOGY

Volume 77 (2021)

Supporting information for article:

Expression and analysis of the SAM-dependent RNA methyltransferase Rsm22 of *Saccharomyces cerevisiae*

Jahangir Alam, Farah Tazkera Rahman, Shiv K. Sah-Teli, Rajaram Venkatesan, M. Kristian Koski, Kaija J. Autio, J. Kalervo Hiltunen and Alexander J. Kastaniotis

S1. Primer designing for Polymerase chain reaction (PCR) amplification of yeast mitochondrial tRNAs

Primers were designed for amplification of the yeast mitochondrial tRNA genes. RNA polymerase promoter site, T7 promoter, was introduced in the 5' ends to achieve DNA templates that can be properly used for *in vitro* transcription. A total of twenty-four sets of primers designed for PCR amplification of the yeast mitochondrial tRNAs were designed. List of primers are given in the ST5.

S2. PCR amplification of yeast mt-tRNAs genes

PCR was done to amplify the yeast mitochondrial tRNA genes. The procedure was carried out using the PTC-100 Peltier effect cycling in a thermal cycler (MJ Research). Isolated DNA from the WT yeast (W1365B) strain was used as a template for the PCR amplification of all mitochondrial tRNAs. The standard protocol from Thermo Scientific Phusion High-Fidelity PCR Kit (user manual no. MAN0013363) was followed for assembling the reaction. The PCR reaction was carried out in thin walled PCR tubes and the mixture contained the following components: 1X reaction buffer, 20 ng template DNA, 25 pmols of forward and reverse primers each, Phusion® High fidelity DNA polymerase, 10 nmol dNTPs and sterile distilled water to adjust the total volume of the reaction up to 50 µl. Special attention was paid while assembling the reaction, that all the reagents were assembled on ice and the enzyme was added in the last step.

S3. Purification of the PCR product

The obtained PCR products were purified using the NucleoSpin® Gel and PCR Clean-up kit, following the protocol in the user manual provided by the manufacturer and stored at -20 °C for forthcoming experiments.

S4. Agarose gel electrophoresis

Agarose gel electrophoresis was used to visualize and analyze the DNA fragments from PCR products, separated according to their molecular weight. Similarly, to identify and analyze the tRNAs obtained by *in vitro* transcription reactions, agarose gel electrophoresis experiments were performed. The DNA fragments from the PCR product was detected by running 5 µl of 1X loading dye on 2% agarose gel. Similarly, for identifying the tRNAs (length ranging from 70-90 bp) a concentration of 1.5-2% agarose gel was used. Ethidium bromide to a final concentration of approximately 0.2-0.5 µg/ml was added to the gel. 100 ng of samples were loaded in the wells to visualize clean bands on the gel. For sizing, Thermo Scientific GeneRuler 1 kb Plus and 100 bp were used as DNA ladders or markers for the experiments. The gel was run in TAE running buffer (40 mM Tris-acetate, 1 mM EDTA) for 1 hour and 30 minutes at a voltage of 80V. The images of the gel were captured with Bio-Rad ChemiDoc XRS+ machine.

S5. *In vitro* transcription of yeast mt-tRNAs

Transcription reaction was assembled following the MEGAscript® Kit (Ambion/Thermo Fisher). The reaction was assembled at room temperature. First, the frozen reagents, RNA polymerase Enzyme mix, 10X reaction buffer and the 4 ribonucleotides solutions (ATP, CTP, GTP and UTP) provided in the kit were thawed. The 10X reaction buffer was kept at room temperature so that the spermidine in the buffer cannot co-precipitate the template DNA. Equal volumes of the four ribonucleotide solutions were mixed to obtain a reaction volume of 20 µl. 300 ng of DNA template was used to achieve an optimal reaction. The amount and the composition of the reagents are listed in the Supplementary table T6. The transcription reaction assembly was mixed gently and incubated at 37 °C for 16 hours. After incubation, the completed transcription reaction was treated with 1 µl TURBO DNase enzyme and incubated for 15 minutes at 37°C for removal of the template DNA. The template is the purified PCR product with T7 promoter for high yield of tRNAs *in vitro*.

S6. Purification of the *in vitro* transcript tRNAs

The transcription reaction mixture was cleaned using the RNeasy® MinElute® Cleanup kit following the basic protocol provided by the manufacturer. In the last step of the cleanup, 14 µl of RNA elute was collected. 1 uL of the RNA sample diluted 10-fold was used to determine the concentration using NanoDrop.

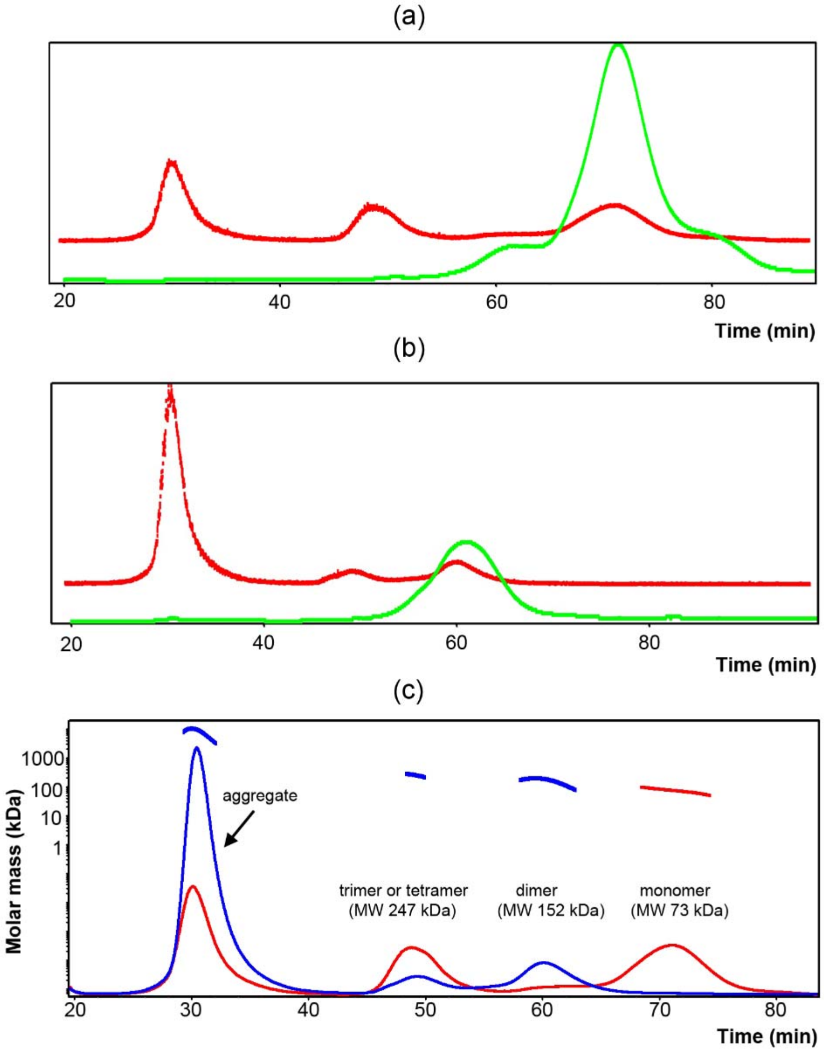

Figure S1 The online SEC-MALS analysis of monomeric (a) and dimeric (b) forms of Sc-Rsm22 using the Äkta system equipped with a Superdex200 10/300GL Increase column (GE Healthcare) and a Wyatt mini DAWN TREOS for recording the light scattering (LS) signal. In the panels a and b only the LS2 (LS signal measured at 90° angle) and UV signals are shown in red and green respectively. The UV signal shows that amount of aggregated protein is very less (less than 5%) in both runs, and the major fraction of the protein sample are either in the monomeric (panel a) or dimeric (panel b) forms. c) The plot of molar mass versus elution time for both monomeric (red) and dimeric (blue) Sc-Rsm22 is shown. Also shown are the corresponding LS signals measured at 90° angle (the same as in panels a and b. The molar masses were calculated from the refractive index (RI) and LS signals using the Astra software.

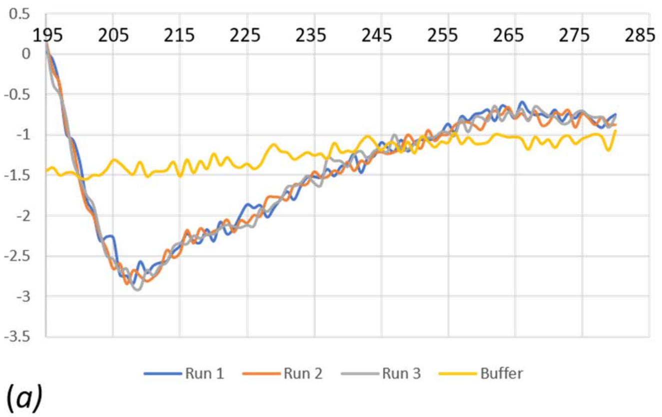

(*a*)

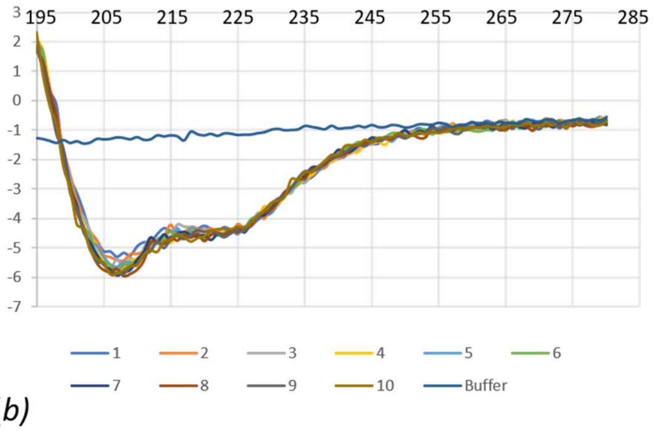

(*b*)

Figure S2 CD spectra: Raw data of CD experiments. Data has been collected in between 280 and 190 nm at 22 °C using a 0.1 cm path-length quartz cuvette. Data in between 190 and 194 nm has not been shown due to the high UV absorbance at low wavelength. (*a*) Presents 3 repetitions of CD spectra of dimeric Sc-Rsm22 including buffer. (*b*) represents 10 repetitions of CD spectra of monomeric Sc-Rsm22 including buffer. Monomeric Sc-Rsm22 subjected to data collection after 30 minutes of the dilution. It shows the monomeric protein became stable after 30 minutes of the dilution.

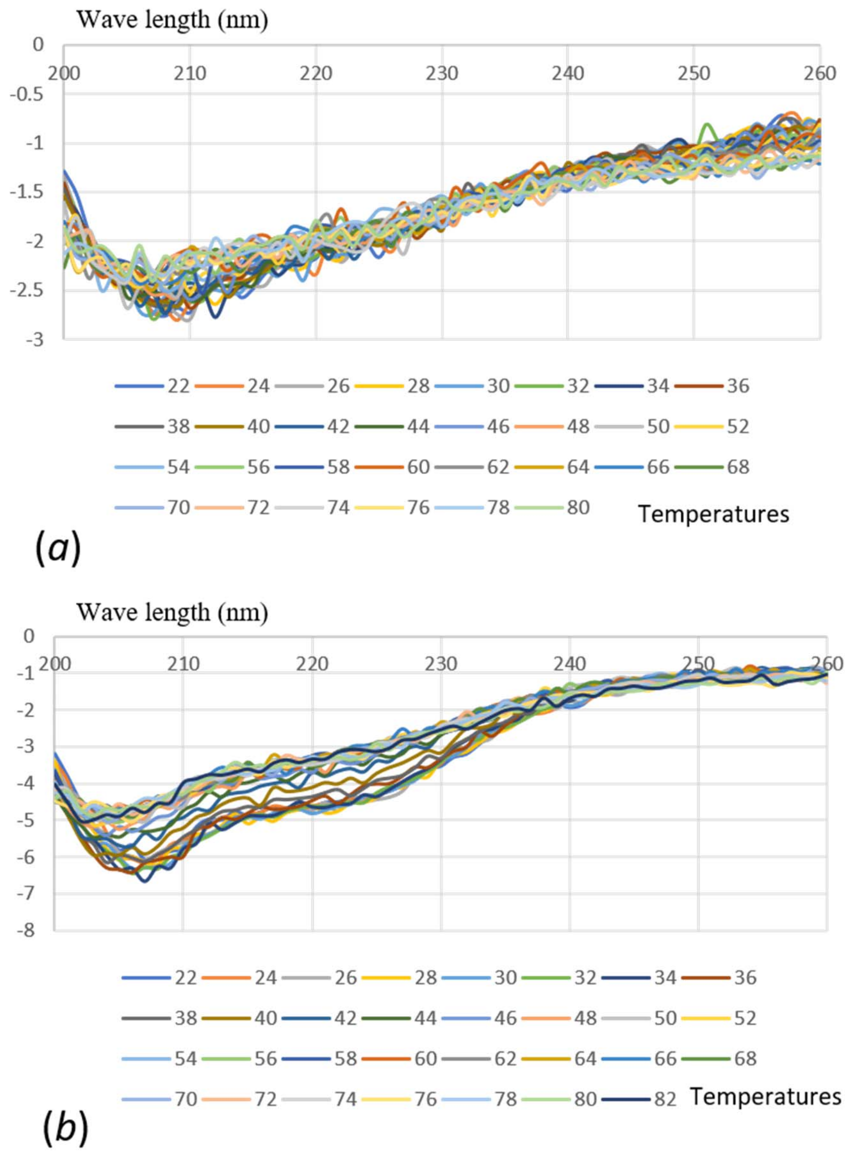

Figure S3 Thermal unfolding: Thermal unfolding was recorded between 190 and 280 nm with a 2 °C step size at 1°C/min ramp rate with ± 0.2°C tolerance (Data in between 200 and 260 nm is shown here). The melting temperature was analyzed with Global3 (Applied Photophysics) using all the recorded data. (a) Thermal unfolding of dimeric Sc-Rsm22. (b) Thermal unfolding of monomeric Sc-Rsm22.

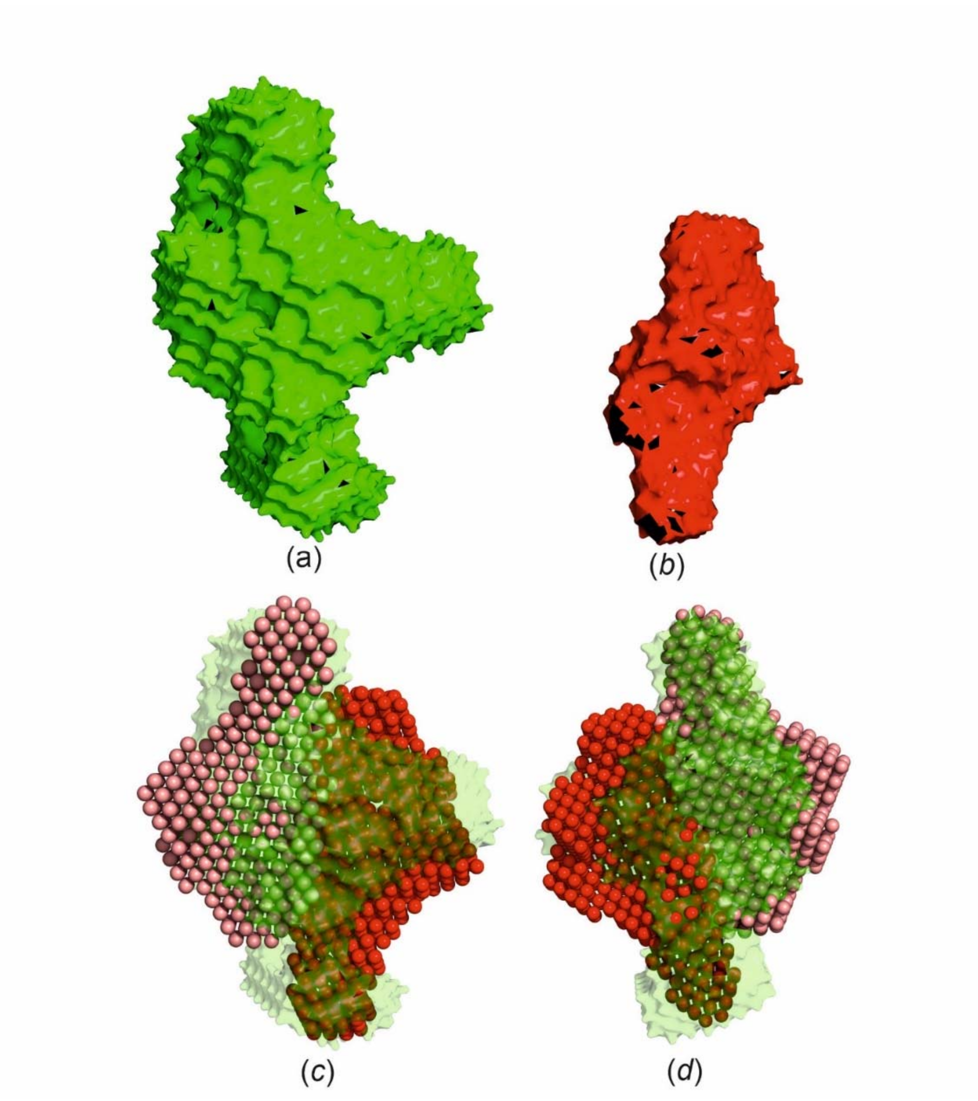

Figure S4 SEC-SAXS (online SAXS) analysis of Sc-Rsm22. (a) The *ab initio* shape of Sc-Rsm22 dimer. (*b*) The *ab initio* shape of Sc-Rsm22 monomer. (*c*) Two identical Sc-Rsm22 monomeric *ab initio* shapes (red and salmon) are superimposed with the *ab initio* model of the dimeric Sc-Rsm22 (green). (*d*) 180 º rotation of superimposed model showed in panel *c*.

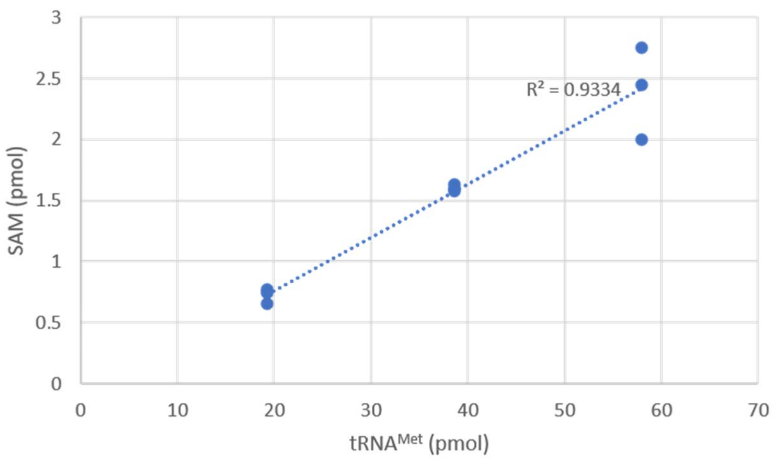

(*a*)

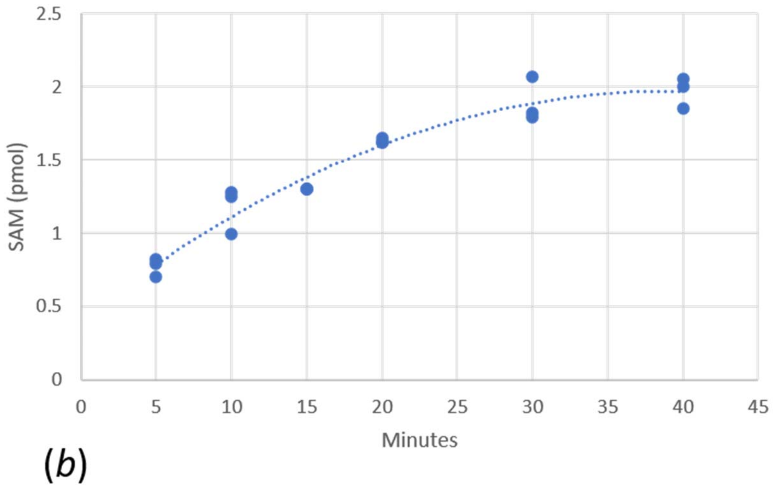

(*b*)

Figure S5 Enzymatic assay of mt-tRNA^{Met} methylation by monomeric Sc-Rsm22. The graphs were generated using MS excel. (*a*) A graph representing methyl group incorporation depending on different concentration of the substrate mt-tRNA^{Met} (pmol). Concentration of enzyme and reaction time were fixed. Graph shows a linear increase of methyl group incorpuration with the increase of amount mt-tRNA^{Met}. (*b*) A graph representing the incorporation of methyl group in relation to the variable duration of reaction. Concentrations of all the reagents including enzyme (Sc-Rsm22) and substrate (32.2 pmol of mt-tRNA^{Met}) were fixed. Graph shows the increase of incopuration methyl group with the increase of reaction time up to 30 minutes.

Table S1 Primer sequences for cloning of *Sc-RSM22* in pET23d plasmid.

Primer type	Sequence (5' to 3')
Forward	TTTTTTCCATGGCTTCAAAGTTCACAAGTTTAAACTTACC
Reverse	TTTTTTCTCGAGTTTTCTATTTACATGTTGTAAAAAATCG

Table S2 Dialysis solvent conditions.

Serial no	Dialysis buffer conditions
1	40 mM Tris pH 7.5, 500 mM NaCl, 5% glycerol
2	40 mM Tris pH 7.5, 200 mM NaCl, 5% glycerol
3	40 mM Phosphate buffer pH 7.5, 500 mM NaCl, 5% glycerol
4	40 mM Phosphate buffer pH 7.5, 200 mM NaCl, 5% glycerol

Table S3 Solvents for DLS experiments.

Serial no	Buffer condition
1	40 mM Hepes pH 7.0
2	40 mM Hepes pH 7.0, 100 mM NaCl
3	40 mM Hepes pH 7.0, 200 mM NaCl
4	40 mM Hepes pH 7.0, 300 mM NaCl
5	40 mM Hepes pH 8.0
6	40 mM Hepes pH 8.0, 100 mM NaCl
7	40 mM Hepes pH 8.0, 200 mM NaCl
8	40 mM Hepes pH 8.0, 300 mM NaCl
9	40 mM MES pH 6.5
10	40 mM MES pH 6.5, 100 mM NaCl
11	40 mM MES pH 6.5, 200 mM NaCl
12	40 mM MES pH 6.5, 300 mM NaCl
13	40 mM MES pH 7.0
14	40 mM MES pH 7.0, 100 mM NaCl
15	40 mM MES pH 7.0, 200 mM NaCl
16	40 mM MES pH 7.0, 300 mM NaCl
17	40mM MOPS pH 6.5
18	40mM MOPS pH 6.5, 100 mM NaCl
19	40mM MOPS pH 6.5, 200 mM NaCl
20	40mM MOPS pH 6.5, 300 mM NaCl
21	40mM MOPS pH 7.5
22	40mM MOPS pH 7.5, 100 mM NaCl
23	40mM MOPS pH 7.5, 200 mM NaCl
24	40mM MOPS pH 7.5, 300 mM NaCl

Table S4 Calculated secondary structure of Sc-Rsm22 in percentage.

Secondary structure	Sc-Rsm22 monomer 1st run	Sc-Rsm22 monomer 2nd run	Sc-Rsm22 Dimer
Helix	34.90 %	30.30 %	27.20 %
Antiparallel	8.20 %	12.40 %	14.20 %
Parallel	8.20 %	8.70 %	9.90 %
Beta-Turn	16.40 %	17.70 %	18.30 %
Random Coil	31.20 %	29.60 %	33.50 %
Total Sum	98.90 %	98.70 %	103.10 %

Table S5 List of the primers used for PCR amplification of yeast mt-tRNA genes. All the forward primers have a T7 promoter sequences (TAATACGACTCACTATAGGG) in the 5' end.

Yeast mitochondrial tRNA (mt-tRNA)	Forward primer sequence after T7 promoter (TAATACGACTCACTATAGGG)	Reverse primer sequence
mt-tRNA^{Ala}	GGGGTTATAGTTAAATTTGGTAGAAC	TATTGGAGTTAATGAGACTTGAACTC
mt-tRNA^{Cys}	GGAGATGTTGTTTTAAGGTTAAAC	TTTAAGAGATGAAGAGAATCGAAC
mt-tRNA^{Asp}	GGATCTGTAGCTTAATAGTAAAG	ATACGAATCTAATCAGATTTG
mt-tRNA^{Glu}	GACCTTATCGTCTAATGGTTACG	TAACCTTAATCGGAATCGAACC
mt-tRNA^{Phe}	GCTTTTATAGCTTAGTGGTAAAGC	TATTGCCCTTAATGAGAATCG
mt-tRNA^{Gly}	ATAGATATAAGTTAATTGGTAAACTGG	AATTATAGATAGCGAGAATCGA
mt-tRNA^{His}	GGTGAATATATTTCAATGGTAGAAA	TAGGGTGAATACTGAGAATCG
mt-tRNA^{Ile}	GAAACTATAATTCAATTGGTTAGAAT	ATATGAAACTAACAGGGATTGA
mt-tRNA^{Lys}	GAGAATATTGTTTAATGGTAAAACAG	TATGAGAATAGCTGGAGTTGAAC
mt-tRNA^{Leu}	GCTATTTTGGTGGAATTGGTA	TATTGCTATTTAAAGGACTTGAACC
mt-tRNA^{Met}	GAGCTTGTATAGTTTAATTGGTTAA	TACTTGTAGAAGGAATTGAACC
mt-tRNA$^{f\text{-}Met}$	TGCAATATGATGTAATTGGTTAAC	TTTATTAGCAATAATACGATTTGAAC
mt-tRNA^{Asn}	GTCCTTATAGCTTATCGGTTAAAGC	GTCCTTAATAGGAATTGAACCC

mt-tRNA^{Pro}	CAGATAGAAGCCAAAAGGTCAG	TCAGATAGGATAGACTCGAACTAAC
mt-tRNA^{Gln}	TGAGTCGTAGACTAATAGGTAAGTTAC	ATATTGAATCGGTTTGATTCG
mt-tRNA^{Arg2}	ATATCTTTAATTTAATGGTAAAATATT	TATAATATCTTATAGGATTTGAACC
mt-tRNA^{Arg1}	GCTCTCTTAGCTTAATGGTTAAAGC	TACTCTCTCCATGATTTGAACATG
mt-tRNA^{Ser2}	GGAAAATTAACTATAGGTAAAGTGG	ATACGGAAAATATGAGATTCGAAC
mt-tRNA^{Ser}	GGATGGTTGACTGAGTGGTTTAAAG	TTACGGATGATGTAGGATTTGAACC
mt-tRNA^{Thr1}	GTAAATATAATTTAATGGTAAAATG	TTTGTAAATACTAAGATTTGAAC
mt-tRNA^{Thr2}	GTTATATTAGCTTAATTGGTAGAGC	TATTGTTATATTAGGGATTTGAACC
mt-tRNA^{Val1}	AGGAGATTAGCTTAATTGGTATAGC	ATTTAGGAAATATAGGGTTCGAACC
mt-tRNA^{Trp1}	AAGGATATAGTTTAATGGTAAAACAG	TATCAAGGATAAAGAGATTCGAAC
mt-tRNA^{Tyr}	GGAGGGATTTTCAATGTTGGTAG	GAAGGGAATAGGAATTGAACCTATG

Table S6 Reagents and assembling of *in vitro* transcription reaction.

Amount	Component
to 20 µl	Nuclease-free Water
2 µl	ATP solution
2 µl	CTP solution
2 µl	GTP solution
2 µl	UTP solution
2 µl	10X Reaction Buffer
1 µl	(optional) UTP as a tracer
1 µl of 0.3 µg/µl template DNA	linear template DNA
2 µl	Enzyme Mix

Table S7 The dpm values of *in vitro* mt-tRNAs methylation by monomeric Sc-Rsm22.

Serial number	Name of tRNA (32.2 pmol)	Average value (average negative value subtracted)	Standard deviation
2	Glu	645	±171
3	Lys	565	±76
4	Leu	552	±55
5	Thr2	752	±33
6	Tyr	732	±46
7	Gln	798	±11
8	Arg1	761	±30
9	Arg2	838	±92
10	Ile	732	±97
11	His	679	±91
12	Phe	850	±33
13	Pro	557	±83
Po14	f-Met	579	±172
15	Ser2	500	±30
16	Trp1	563	±103
17	Thr1	630	±21
18	Gly	801	±23
